# Validating the effectiveness of an AI algorithm for pulmonary tuberculosis screening using chest X-ray: Retrospective study and test accuracy with localizer images of the chest CT

**DOI:** 10.1371/journal.pone.0338810

**Published:** 2026-02-27

**Authors:** Yixiao Wei, Xiaojing Cui, Lingtao Chong, Chunlei Wang, Min Liu, Xiaoliang Chen, Lintao Zhong

**Affiliations:** 1 National Clinical Research Center of Respiratory Diseases, Center for Respiratory Diseases, China-Japan Friendship Hospital, Beijing, China; 2 National Center for Respiratory Medicine, China-Japan Friendship Hospital, Beijing, China; 3 Institute of Respiratory Medicine, Chinese Academy of Medical Sciences, China-Japan Friendship Hospital, Beijing, China; 4 Department of Pulmonary and Critical Care Medicine, China-Japan Friendship Hospital, Beijing, China; 5 Fuwai Hospital, National Center for Cardiovascular Diseases, Chinese Academy of Medical Sciences and Peking Union Medical College, Beijing, China; 6 Laboratory of Clinical Microbiology and Infectious Diseases, China-Japan Friendship Hospital, Beijing, China; 7 Department of Radiology, China-Japan Friendship Hospital, Beijing, China; 8 Nosocomial Infections Management Office, China-Japan Friendship Hospital, Beijing, China; Chulalongkorn University Faculty of Medicine, THAILAND

## Abstract

**Introduction:**

China accounted for 6.8% of global TB cases, and most patients are first diagnosed in general hospitals where chest X-rays (CXR) are widely used for early TB detection. To facilitate diagnosis in resource-limited settings, our study evaluates a CNN-based AI model trained on Chinese CXR data (JF CXR-1 v2), including its experimental application to CT localizer images.

**Materials and methods:**

This retrospective study was conducted at China-Japan Friendship Hospital, including 290 CXR images and 433 CT localizer images from TB patients diagnosed between 2017 and 2021. The AI algorithm’s diagnostic performance was assessed using sensitivity, specificity, accuracy, Kappa value, and AUC from ROC analysis.

**Results:**

The AI algorithm demonstrated high diagnostic performance on CXR images, achieving an AUC of 0.960 with 91.7% sensitivity and 92.7% specificity in bacteriologically confirmed TB cases. On localizer images of the chest CT, while the performance was more modest (AUC 0.719), a significant correlation between CXR and CT predictions in 105 paired cases suggests potential for cross-modality application with further validation.

**Discussion & conclusions:**

The algorithm shows decent diagnostic capability for the CXR samples in this study. This AI algorithm developed based on CXR can, to some extent, identify the imaging features of pulmonary TB when applied to localizer images of chest CT.

## Introduction

According to the World Health Organization’s (WHO) Global Tuberculosis Report 2024, thirty high pulmonary tuberculosis (TB) burden countries accounted for 87% of the world’s TB cases in 2023, with China accounting for 6.8% [1]. In China, diagnosed TB patients are referred from general hospitals to specialized infectious disease hospitals for treatment by TB experts. Since the initial symptoms of TB, such as prolonged cough, are nonspecific and common to many respiratory diseases, most patients first consult general hospitals. This situation applies to the majority of countries worldwide.

Chest X-ray (CXR) plays an important role in the early detection of pulmonary TB. In high-burden developing countries, it helps identify TB early among people with respiratory symptoms and can also detect subclinical TB during routine health screenings [2]. In developed countries like the United States, CXR is recommended for people who test positive on IGRA or TST to check for active TB [3]. However, TB can show up in many different ways on CXR, which makes it hard to recognize, especially for physicians and radiologists who are not familiar with its typical signs. Fortunately, some of these features are recognizable by machines, making CXR a good tool for AI-based TB detection, especially in areas with limited medical resources. Pulmonary TB shows diverse radiologic patterns, such as consolidation, cavitation, nodules, lymphadenopathy, pleural effusion, and miliary disease. Any lung lobe may be affected, although lower-lobe involvement is relatively more common in primary TB, in contrast, reactivation TB typically affects the upper lobes, with occurring in approximately 50% of cases [4]. Moreover, CXR are also cheaper, faster, easy to implement, and are available in more primary care hospitals.

Computer-aided detection software utilizing artificial intelligence (AI) systems may offer a solution to help general physicians in general hospitals quickly screen for TB patients. Over the past decade, research on using machine learning techniques to analyze CXR images for screening cardiopulmonary abnormalities has experienced explosive growth [5]. The advent of convolutional neural network (CNN)-based deep learning (DL) provides the basis for imaging-based Artificial Intelligence (AI) solutions [6,7]. The performance of DL-based medical imaging tools for TB screening has been improving since 2016, driven by the expansion of datasets and the introduction of ensemble deep neural networks (DNN) and DL-based algorithms [8]. In March 2021, the World Health Organization (WHO) updated its Systematic Screening for TB guidelines, which, for the first time, recommended the use of CAD4TB as an alternative to human interpretation of digital chest X-rays [9]. In this context, we aim to train a new model using recent CXR images of TB patients from China and evaluate it with images from patients in Chinese general hospitals.

JF CXR-1 v2 (JF Healthcare, Jiangxi, China), an AI software had been developed based on CXR and built on a convolutional neural network algorithm. This is a simultaneous CXR computer-aided detection (CAD) software based on CNNs that detects multiple thorax diseases, such as TB, lung mass, and lung nodules. The software was trained on CXRs from township-level hospitals across China. However, its accuracy in detecting TB among more complex patient populations seen in tertiary general hospitals remains to be evaluated and improved. Therefore, in this study, we aimed to validate the effectiveness of the pulmonary TB imaging screening algorithm using CXR images from diagnosed TB patients at China-Japan Friendship Hospital (CJFH), the National Center for Respiratory Medicine of China.

CT (Computed Tomography) is an imaging tool that combines X-rays with computer technology to produce more detailed, cross-sectional images of the body. In general hospitals in China, CT is more commonly utilized for TB screening and diagnosis [10,11]. Since the imaging principles are similar, and CT CAD for TB remains underdeveloped [12], we made an attempt to explore the use of CXR algorithm for analyzing localizer images of chest CT to temporarily bridge the gap. These images are not diagnostic scans and have limited resolution and coverage, and the exploratory analysis was designed as a hypothesis-generating study.

## Materials and methods

### Operational mechanism of the TB screening algorithm

The algorithm divides each CXR into grids and uses radiologist-provided lesion annotations to label grid cells during training. A deep learning model then predicts TB probability per grid and aggregates these to yield an overall TB score. Detected lesions are visualized using density smoothing. Further details are provided in this section.

#### Algorithm overview.

**Fig 1:**
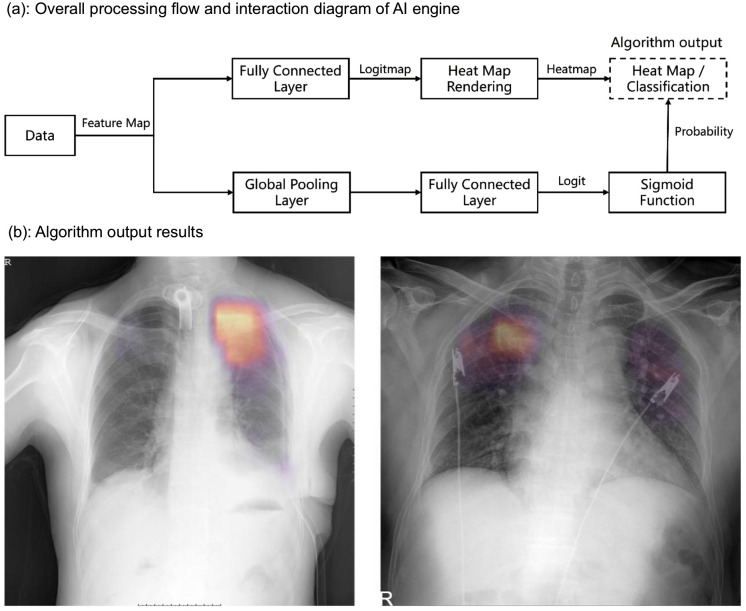
Overall workflow and algorithm output.

We use deep learning methods to predict the probability of a patient having active pulmonary TB based on chest X-rays and visualizes the lesion locations using heatmaps. This method designs a dual-path processing based on CNN. The flowchart is shown in Fig 1(a).

The input data is preprocessed and transformed into feature maps, which are then used in two parallel processes: one generates heatmaps to highlight lesion areas, and the other computes a probability score for active TB through global pooling, a fully connected layer, and a sigmoid function. The output result after algorithm processing is shown in Fig 1(b).

#### Model architecture and training process.

**Fig 2:**
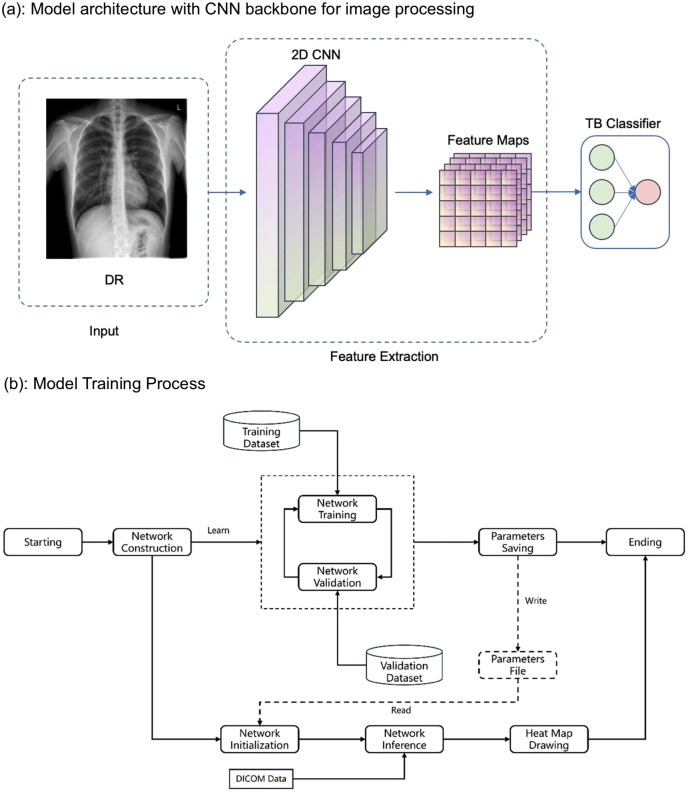
Model architecture and training workflow.

**Fig 3:**
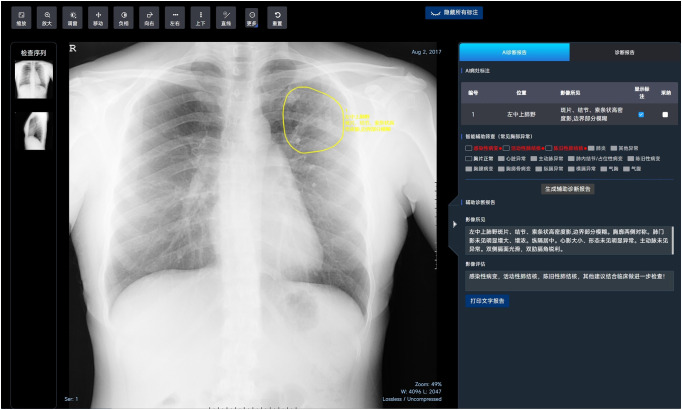
An example of the algorithm interface. The upper toolbar contains basic image viewing functions such as zooming, window adjustment, panning, inversion, rotation, and measurement tools. The left panel displays the examination series, while the central window shows the chest image for AI analysis and visualization. The highlighted region in yellow in the left upper lung field demonstrates patchy, nodular, and linear high-density opacities with partially blurred margins. The right panel presents the AI diagnostic report, including three major sections: (1) AI lesion annotation: displays the detected lesion location and imaging findings (e.g., patchy, nodular, and linear high-density opacities in the left upper lung field). (2) Intelligent auxiliary screening: lists common thoracic abnormalities identified by the algorithm; the red indicators highlight positive findings for infectious lesions, active pulmonary TB, and inactive pulmonary TB. (3) Auxiliary diagnostic report: provides the textual interpretation and summary, including imaging description (Imaging findings) and overall assessment (Imaging evaluation).

This model employs a CNN as the backbone network to process input images, as shown in Fig 2(a). The training process of this model is shown in Fig 2(b), which is mainly divided into two major processes. The first process involves completing the model training based on training data and validation data, and designing storage conditions to save the model files according to requirements. Radiologists annotated lesion areas in the training images, and these annotations were converted into binary masks (1 for lesions, 0 for background) as training labels. The model learned by minimizing the loss between predicted probability maps and ground-truth masks. The second process is the testing process, where the trained model files are loaded into the model, DICOM files are inputted into the model, the model completes data processing and forward inference, outputs prediction results (a probability score ranging from 0 to 1 indicating the likelihood of active TB), and draws a heatmap. An example of the algorithm interface is shown in Fig 3, illustrating how image analysis results are displayed.

The model was trained using a batch size of 12, the Adam optimizer, and the binary cross-entropy loss function, with a learning rate of 0.0001. A hold-out validation strategy (9:1 train/validation split) was adopted to monitor model convergence.

The JF CXR-1 algorithm was developed using 14,160 CXRs from township-level hospitals throughout China, representing diverse patient populations and radiographic findings. Its performance was externally validated on an independent dataset of 13,122 CXRs, achieving an AUC of 0.94, sensitivity of 0.91, and specificity of 0.81, which is consistent with WHO-recommended benchmarks for TB screening.

### Study design and methods

A retrospective study was conducted. Patients diagnosed with TB following China’s National TB Diagnosis Guideline and reported to Chinese Center for Disease Control and Prevention from 2017 to 2021 in National Center for Respiratory Medicine of China were retrospectively included. Another group of healthy individuals undergoing annual wellness visit at the same hospital were used as the control group. The algorithm was run to diagnose active TB according to images of CXR. We measured the sensitivity, specificity, consistency rate, and Kappa value for the diagnosis, and also plotted the ROC curve. For the localizer images of chest CT’s test, the same measurement methods were applied. This study focuses on the predictive accuracy of active TB, but the AI also outputs predictions for other abnormalities, such as inactive TB and pneumonia, which will be discussed in detail later.

Ethics statement: The studies involving humans were approved by The Ethics Committee of China-Japan Friendship Hospital. The studies were conducted in accordance with the local legislation and institutional requirements. Written informed consent for participation in this study was provided by the participants’ legal guardians/next of kin. No potentially identiﬁable images or data are presented in this study.

#### Study setting and population.

This retrospective study was conducted at the China-Japan Friendship Hospital, a tertiary care center specializing in respiratory medicine. Although it is not a designated hospitals for TB in China, a considerable number of TB cases are diagnosed when seeking treatment for unspecific symptoms or other diseases. Newly diagnosed TB patients at this hospital will be reported to Chinese Center for Disease Control and Prevention (CCDC).

Participants were recruited from 1678 patients newly diagnosed with TB following the 2017 China’s National TB Diagnosis Guideline (WS288–2017) [13] at CJFH and reported to CCDC from 2017 to 2021. According to the guideline, TB cases are classified into three categories: (1) Bacteriologically confirmed TB, defined as patients with positive microbiological evidence of Mycobacterium tuberculosis infection, including smear microscopy, culture, or molecular (nucleic acid) assays. (2) Clinically diagnosed TB, defined as cases with compatible clinical, radiological, and epidemiological findings but without bacteriological confirmation, where anti-TB treatment was initiated based on comprehensive clinical judgment. (3) Suspected TB, defined as patients with suggestive symptoms or imaging features who did not yet meet the above criteria but required further evaluation. Detailed diagnostic criteria are described in WS 288–2017, available on the official National Health Commission website (nhc.gov.cn).

The inclusion criteria were “received the CXR examination within 1 year before diagnosis or could provide the image (DICOM format) of the posterior-anterior CXR taken within 1 year before TB diagnosis”. Given that TB often develops gradually over months, this one-year window was selected to capture early radiographic manifestations preceding clinical diagnosis [14]. The exclusion criteria were “subjects whose CXR images do not meet the diagnostic requirements”. This study was approved by the ethics committees of China-Japan Friendship Hospital. The imaging files were accessed on April 10, 2024. The investigators did not have access to any personally identifiable information (such as names, ID numbers, or hospital admission numbers) during or after data collection.

#### Procedures.

CXR images of participants before standard TB treatment was exported from the Electronic Medical Records system, saved in DICOM format. For cases with multiple images under the same case number, the record with the highest AI-predicted TB value was selected. A small number of cases with case number mismatches were excluded. After data cleaning, a total of 290 valid CXR images were obtained. The average age of TB patients is 54.56 years, with 166 male patients (57.24%) and 124 female patients (42.76%). A group of healthy individuals undergoing annual wellness visit at the same hospital in 2023 with radiological diagnosis of “no significant abnormalities detected” were randomly extracted and used as the control group. A total of 315 valid CXR images were obtained. The average age of the control group is 34.71 years, with 146 male patients (46.35%) and 169 female patients (53.65%).

For the localizer images of chest CT’s test, among the 1,678 TB diagnosed participants, 433 valid localizer images were obtained as positive samples. The average age was 47.88 years, with 270 male patients (62.36%) and 163 female patients (37.64%). Since the algorithm needed to incorporate more negative sample, 166 chest CT images from patients who visited the fever clinic in early 2023 were retrieved. These patients had been diagnosed with “pulmonary infection” by radiologists. The fever clinic is a dedicated outpatient service for patients with upper respiratory infections, specifically established by Chinese hospitals during the pandemic. The control group had an average age of 61.89 years, with 106 male patients (63.86%) and 60 female patients (36.14%). Unlike healthy CXRs, which can be readily collected from the annual wellness visit population, CT examinations are generally performed for patients with respiratory symptoms. As a result, obtaining completely healthy CT images is challenging. In this study, we used cases with mild pulmonary infections as negative controls for CT localizer analysis. While this approach reflects real-world clinical scenarios, it may have reduced the apparent specificity of the algorithm.

**Fig 4:**
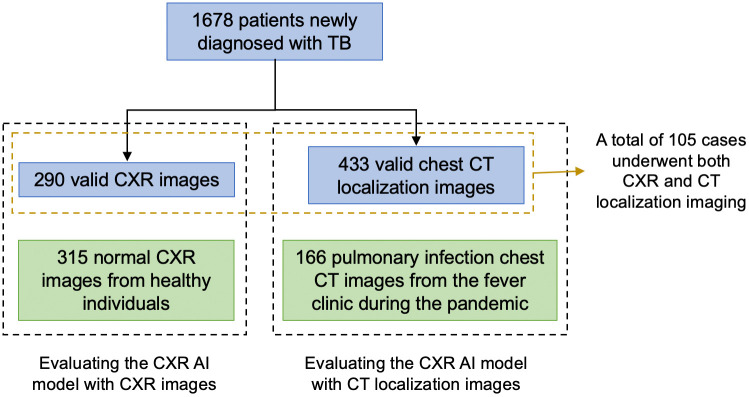
The images collecting process and the evaluation workflow.

We input the DICOM format files into the AI-based CAD software (JF CXR-1 v2, produced by Jiangxi Zhongke Jiufeng Smart Medical Technology Co., Ltd.). The AI algorithm produced a probability score for each anonymized image to predict its likelihood of being TB-positive. The images collecting process and the evaluation workflow are shown in Fig 4. The analysis code and data used in this study are available in the Supporting Information as S1 File.

#### Data analysis.

The primary evaluation metrics were sensitivity and specificity, while the secondary metric was the area under the receiver operating characteristic (ROC) curve (AUC). We also used accuracy and Kappa value to evaluate the performance. A patient’s image diagnosed by the AI software as TB or non-TB was considered a true positive (TP) or false negative (FN) if the patient had TB, and a false positive (FP) or true negative (TN) if the patient did not have TB. Sensitivity was calculated as TP/ (TP + FN), specificity as TN/ (TN + FP). Accuracy was calculated as (TP + TN)/Total Number of Cases. κ (Kappa value) was calculated as $\frac{\text{2}\left[(TP\times TN)-(FP\times FN)\right]}{\left(TP+FP)(FP+TN)+(TP+FN)(FN+TN\right)}$. ROC curve was plotted with sensitivity on the Y-axis and 1-specificity on the X-axis, with the AUC representing the area under this curve. Model thresholds were determined based on post-hoc performance optimization in this dataset, and no adjustment was made for demographic factors such as age or sex.

#### Role of the AI developers.

The AI developer had no role in study design, data collection, analysis, or manuscript writing, but they provided us with a free account to use the software and free technical support.

## Results

### Chest X-Ray images

**Fig 5:**
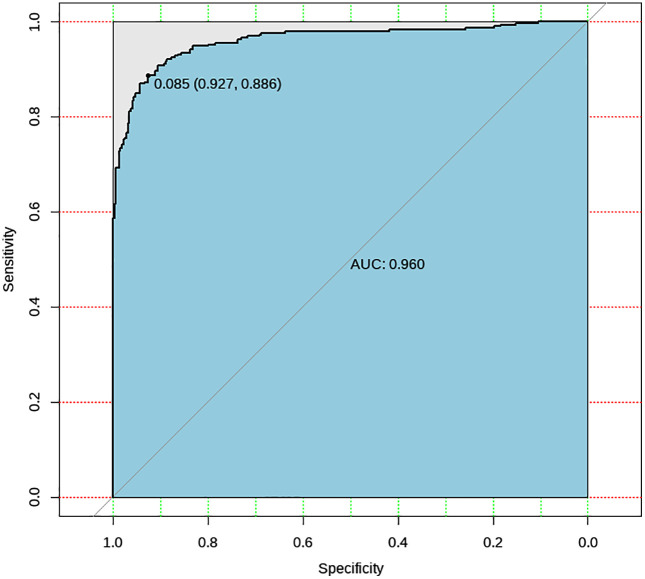
ROC curve of the TB Screening Al Algorithm (CXR). The values in parentheses shown on the ROC curves represent the x- and y-coordinates of the selected operating points.

Plot the ROC curve of AI prediction results, as shown in Fig 5. According to the principle of maximizing the area under the ROC curve (AUC), we determined the optimal threshold, i.e., the cutoff value among the prediction results output by the algorithm that achieves the best balance between sensitivity and specificity. In the TB patients’ CXR dataset, the optimal threshold for the AI model was 0.085.

Based on different thresholds, the accuracy metrics and their 95% confidence intervals were calculated. We display accuracy metrics for two thresholds: 0.350, which was the best performing threshold in the previous algorithm’s testing phase (AUC = 0.960, 95% CI 0.945–0.976), and 0.085, which is the optimal threshold identified in this study (AUC = 0.960, 95% CI 0.945–0.976), with the results shown in Table 1.

**Table 1:** Accuracy metrics of the TB Screening AI Algorithm (CXR). Threshold = 0.350 represents the best-performing threshold identified in the previous version of the algorithm, while threshold = 0.085 was determined as the optimal cutoff in the current study.

Threshold	Accuracy	Sensitivity	Specificity	AUC	Kappa Value
0.350	90.7% (90.7%–90.8%)	66.9% (61.5%–72.3%)	99.4% (98.5%–100.0%)	0.960 (0.945–0.976)	0.814 (0.768–0.861)
0.085	90.7% (90.7%–90.8%)	88.6% (85.0%–92.3%)	92.7% (89.8%–95.6%)	0.960 (0.945–0.976)	0.814 (0.768–0.861)

In addition to outputting the predicted probability of active TB, the AI also generates predictions for other abnormalities, including inactive TB, pneumonia, pneumothorax, nodules/masses, pleural lesions, and cardiomegaly. We categorize the prediction results into four groups: active TB, inactive TB, other abnormalities, and no abnormalities. The proportion of predictions is analyzed for each diagnostic outcome, as shown in Table 2.

**Table 2:** Accuracy by diagnostic outcomes.

Diagnostic Outcomes Classification	# of Cases	Active TB	Inactive TB	Other Abnormalities	No Abnormalities	Proportion of Active TB	Proportion of Abnormal Cases
Bacteriologically Confirmed Cases	145	133	0	6	6	91.7%	95.9%
Clinically Diagnosed Cases	140	121	0	5	14	86.4%	90.0%
Suspected Cases	5	3	0	1	1	60.0%	80.0%
Summary	290	257	0	12	21	88.6%	92.8%
On the 145 bacteriologically confirmed cases and 315 normal CXRs, AI yielded the following performance:							
**Bacterio-confirmed TB (Positive)**	**AI Predicts TB**			**AI Predicts Normal**		**Total**	
**Normal (Negative)**	133			12		145	
**Total**	23			292		315	
	156			304		460	

Sensitivity = 133/ (133 + 12) = 91.7%. Specificity = 292/ (292 + 23) = 92.7%.

To align with previous CAD models evaluation studies, such as an study on a WHO recommended screening tool CAD4TB in 2020 [15], which uses Xpert-confirmed TB cases as the reference standard, we re-evaluated our algorithm using only the 145 bacteriologically confirmed cases as true positives, and 315 normal CXRs as true negatives. As shown in Table 2, under the optimal threshold (0.085), our model correctly identified 133 out of 145 confirmed TB cases (sensitivity = 0.917) and 292 out of 315 normal cases (specificity = 0.927). This performance demonstrates competitive diagnostic accuracy comparable to the latest versions of CAD4TB.

### Localizer images of chest CT

**Fig 6:**
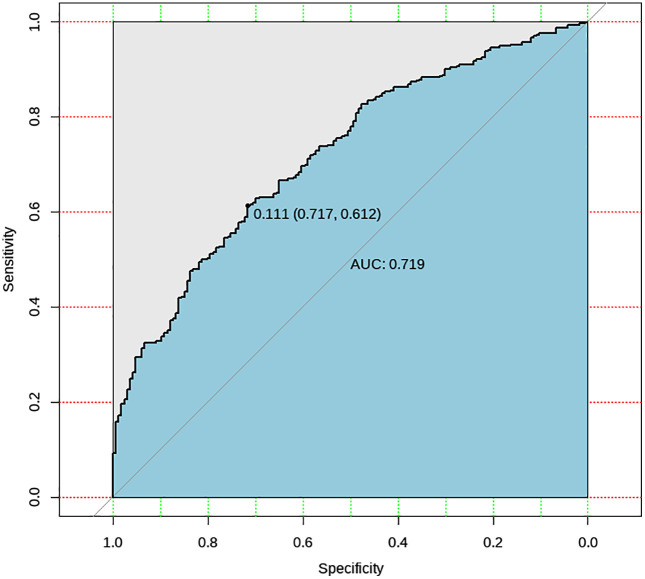
ROC curve of the TB Screening Al Algorithm (localizer images of chest CT). The values in parentheses shown on the ROC curves represent the x- and y-coordinates of the selected operating points.

The ROC curve of the AI prediction results is shown in Fig 6. In the TB patients’ localizer images dataset, the optimal threshold for the AI was 0.111, with a calculated sensitivity of 0.612 and specificity of 0.717.

Based on different thresholds, the accuracy metrics and their 95% confidence intervals were calculated. We display accuracy metrics for two thresholds: 0.350, which was the best performing threshold in the previous algorithm’s testing phase (AUC = 0.719, 95% CI 0.675–0.763), and 0.111, which is the optimal threshold identified in this study (AUC = 0.719, 95% CI 0.675–0.763). with the results shown in Table 3.

**Table 3:** Accuracy metrics of the TB Screening AI Algorithm (Localizer Images of Chest CT). Threshold = 0.350 represents the best-performing threshold identified in the previous version of the algorithm, while threshold = 0.111 was determined as the optimal cutoff in the current study.

Threshold	Accuracy	Sensitivity	Specificity	AUC	Kappa Value
0.350	64.1% (64.0%–64.2%)	16.6% (13.1%–20.1%)	98.8% (97.1%–100.0%)	0.719 (0.675–0.763)	0.269 (0.198–0.339)
0.111	64.1% (64.0%–64.2%)	61.2% (56.6%–65.8%)	71.7% (64.8%–78.5%)	0.719 (0.675–0.763)	0.269 (0.198–0.339)

### Cases underwent both CXR and CT localization imaging

**Fig 7:**
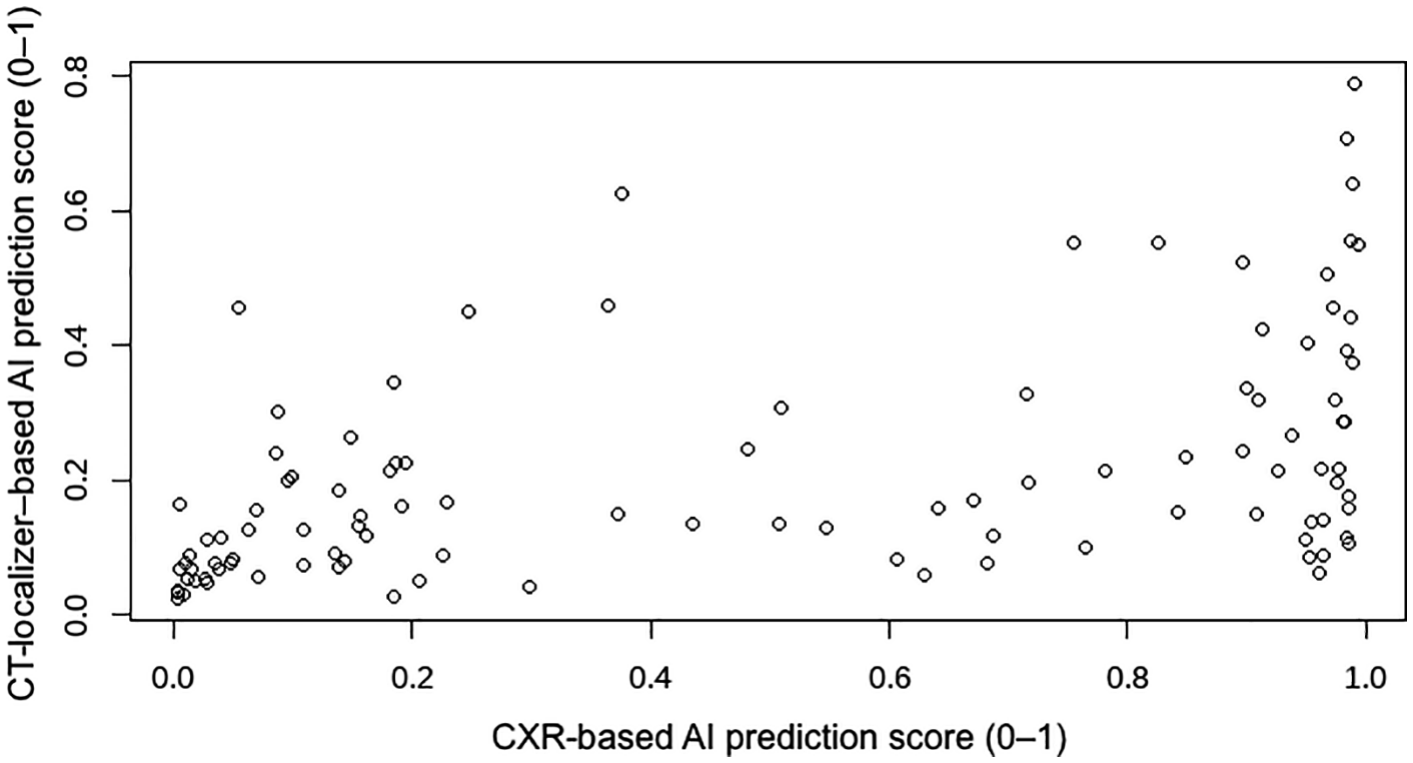
Scatter plot of Al performance in CXR and localizer images of the chest CT in the 105 cases. Scatterplot showing the correlation between CXR-based and CT-localizer–based AI prediction scores for 105 tuberculosis cases. Each point represents the AI-inferred probability (0–1) of active TB for each patient. A significant correlation was observed (Spearman r = 0.577, P < 0.001).

The localizer images of the chest CT group used pulmonary infection CTs as the negative samples, which may underestimate the algorithm’s performance compared to using healthy controls as the negative samples. We identified 105 pulmonary TB cases that underwent both CXR and CT localization imaging. Spearman correlation showed a significant association (r = 0.577, p = 0). The scatter plot is shown in Fig 7.

**Fig 8:**
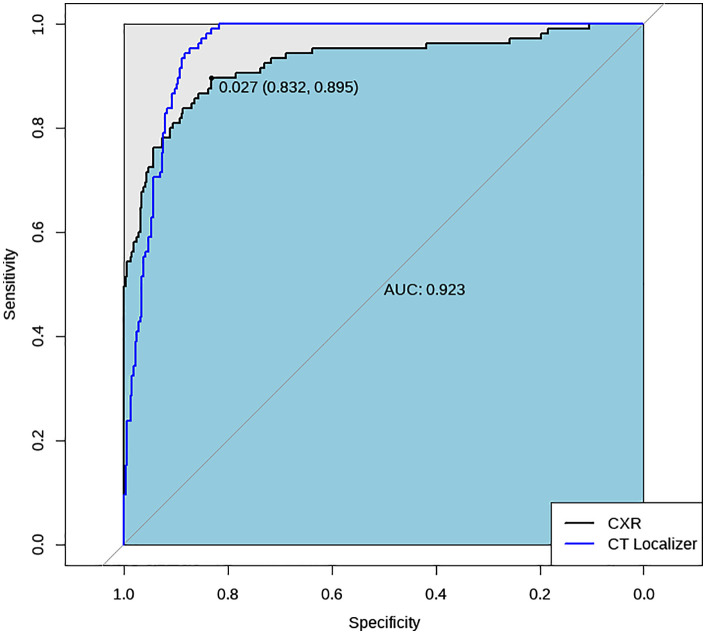
ROC curves of CXR and localizer images of the chest CT in the 105 cases. The values in parentheses shown on the ROC curves represent the x- and y-coordinates of the selected operating points.

Among the 105 patients, since the healthy controls used in the CXR analysis did not undergo CT scans, their localizer images of the chest CT predictions could not be assessed. Assuming these negative samples had localizer images of the chest CT, and their AI predictions were identical to those from CXR, a comparison of ROC curves between CXR and localizer images of the chest CT can be made, as shown in Fig 8. Based on Youden’s index, the optimal threshold for CXR was 0.027, yielding a sensitivity of 0.895 and a specificity of 0.832. For localizer images of the chest CT, the optimal threshold was 0.041, with a sensitivity of 0.952 and specificity of 0.873.

Accuracy metrics and their 95% confidence intervals for CXR and localizer images of the chest CT at different thresholds are shown in Tables 4 and 5. We display accuracy metrics for two thresholds in Table 4: 0.350, which was the best performing threshold in the previous algorithm’s testing phase (AUC = 0.923, 95% CI 0.889–0.958), and 0.027, which is the optimal threshold identified in this study (AUC = 0.923, 95% CI 0.889–0.958). We display accuracy metrics for two thresholds in Table 5: 0.350, which was the best performing threshold in the previous algorithm’s testing phase (AUC = 0.953, 95% CI 0.935–0.971), and 0.041, which is the optimal threshold identified in this study (AUC = 0.953, 95% CI 0.935–0.971).

**Table 4:** Accuracy metrics of the TB Screening AI Algorithm in the 105 Cases (CXR). Threshold = 0.350 represents the best-performing threshold identified in the previous version of the algorithm, while threshold = 0.027 was determined as the optimal cutoff in the current study.

Threshold	Accuracy	Sensitivity	Specificity	AUC	Kappa Value
0.350	84.8% (84.7%–84.8%)	54.3% (44.8%–63.8%)	99.4% (98.5%–100.0%)	0.923 (0.889–0.958)	0.641 (0.563–0.720)
0.027	84.8% (84.7%–84.8%)	89.5% (83.7%–95.4%)	83.2% (79.0%–87.3%)	0.923 (0.889–0.958)	0.641 (0.563–0.720)

**Table 5:** Accuracy metrics of the TB Screening AI Algorithm in the 105 Cases (Localizer Images of Chest CT). Threshold = 0.350 represents the best-performing threshold identified in the previous version of the algorithm, while threshold = 0.041 was determined as the optimal cutoff in the current study.

Threshold	Accuracy	Sensitivity	Specificity	AUC	Kappa Value
0.350	89.3% (89.2%–89.3)	18.1% (10.7%–25.5%)	99.4% (98.5%–100.0%)	0.953 (0.935–0.971)	0.743 (0.673–0.812)
0.041	89.3% (89.2%–89.3%)	95.2% (91.2%–99.3%)	87.3% (83.6%–91.0%)	0.953 (0.935–0.971)	0.743 (0.673–0.812)

Because the two datasets consisted of independent populations, we compared the AUCs using the unpaired DeLong test. 95% confidence intervals (CIs) were computed based on the DeLong method. A P-value < 0.05 was considered statistically significant. The AI algorithm demonstrated an AUC of 0.923 (95% CI, 0.889–0.958) for CXR images and 0.953 (95% CI, 0.935–0.971) for localizer images of the chest CT. According to the unpaired DeLong test, the difference in AUCs (ΔAUC = −0.03) was not statistically significant (P = 0.135).

It is worth noting that the current AUC value for localizer images of the chest CT is likely overestimated, as the predicted scores for negative samples were derived from CXR-based predictions; in reality, these negative cases did not undergo CT localization imaging. However, based on empirical evidence, AI models tend to perform well in identifying negative samples. Therefore, the predictive potential of localizer images of the chest CT remains promising and warrants further investigation.

## Discussion and limitations

### Discussion

Compared to previous CAD models, our AI is designed for complex hospital patients and trained on Chinese CXRs, showing better regional adaptation and diagnostic accuracy. Previous CAD models were predominantly used for large-scale screenings of generally healthy populations [16,17]. Among them, CAD4TB v6 (Delft Imaging), Lunit Insight CXR (Lunit Insight), and qXR v2 (Qure.ai) were assessed by the WHO to inform its CAD recommendations in TB screening guidelines [18]. Notably, CAD4TB has been widely adopted, now available in over 40 countries and having screened nearly 8 million people worldwide [9]. CAD4TB v6, in particular, demonstrated its efficacy as a pre-screening tool in high-burden settings, achieved a specificity of 76% obtained at a fixed 90% sensitivity with the Xpert reference standard [15]. In contrast, our new AI tool evaluated in this study is designed for use in general hospital environments, where patients typically present with more complex underlying conditions. Moreover, this model was trained on CXRs from Chinese patients, making it more region-specific compared to previous CAD tools. Last but not least, re-evaluation of our algorithm using bacteriologically confirmed cases as the reference standard yielded a sensitivity of 91.7% and specificity of 92.7%, indicating diagnostic performance on par with CAD4TB.

In high TB burden areas, Computer-aided detection software can compensate for the shortage of radiologists in primary care clinics, identifying patients who need medical intervention [19,20]. In general hospitals in countries where TB is prevalent, AI-assisted diagnosis can help doctors identify TB patients more quickly, facilitating a more rational arrangement of TB-related pathogen testing [21,22]. From a health economics perspective, this can help reduce medical costs. CAD technologies have the potential to TB screening and help close the gap in identifying nearly three million people with TB who go undiagnosed each year globally [23–25].

According to WHO, an effective tuberculosis screening tool must achieve either optimal performance characteristics (95% sensitivity and 80% specificity) or at least the minimum acceptable thresholds (90% sensitivity and 70% specificity) [26]. Our AI algorithm demonstrates a sensitivity of 0.886 and a specificity of 0.927 using TB cases reported to the CCDC as the reference standard at the optimal threshold of 0.085, with re-evaluation using bacteriologically confirmed cases as the reference standard yielding a sensitivity of 0.917 and specificity of 0.927. High sensitivity is critical for screening tests, as the primary objective is the early detection of TB disease. However, if specificity is low during the screening stage, a considerable proportion of individuals without TB disease will be referred for further screening or diagnostic evaluation, leading to increased costs. From this perspective, this algorithm can either focus on maximizing case detection or prioritizing efficiency, depending on the specific implementation goals. The JF CXR-1 v2 is also one of the CAD products evaluated in a 2024 external validation study on AI-assisted TB detection in South Africa, demonstrating an AUC between 0.8 and 0.9, which indicates good accuracy when compared to microbiological evidence. In threshold analysis, JF CXR-1 v2, along with three other algorithms, maintained a high sensitivity (>90%) across a wide range of thresholds. However, like other CAD products, JF CXR-1 v2’s performance showed a decline in older individuals, those with a history of tuberculosis, and people with HIV, highlighting the challenges faced in these subpopulations [27]. JF CXR-1 v2’s statistical metrics are satisfactory, but further refinement and upgrades are needed in the future. In the next stage, we can analyze the imaging characteristics of the specific samples and optimize the AI algorithm accordingly.

Additionally, stratified data on bacteriologically confirmed cases, clinically diagnosed cases, and suspected cases shown in Table 2 indicate that JF CXR-1 v2 has the potential to contribute to the detection of TB among people either with or without bacteriological confirmation. The AI algorithm classified 91.7% of Bacteriologically Confirmed Cases and 86.4% of Clinically Diagnosed Cases as Active TB. It’s important to note that the microbiological detection of TB is critical, because it enables accurate diagnosis and ensures that the most effective treatment regimen tailored to the drug resistance profile can be initiated as early as possible. Although there are now molecular diagnostic methods for TB, such as Xpert, which help address the long turnaround time associated with culture-based diagnosis, obtaining microbiological evidence is not always possible, particularly in the early stages of the disease when bacterial load is low or when lesions are located in inaccessible areas [28,29]. Overall, the proportion of bacteriologically confirmed TB cases remains relatively low. Of the 6.9 million people diagnosed with pulmonary TB worldwide in 2023, only 62% were bacteriologically confirmed [1]. The algorithm’s predictions may help address a key challenge in TB diagnosis by enabling early diagnostic expectation before bacteriological confirmation and offering an additional reference in clinically diagnosed cases.

We have noticed that several CT CAD TB-related articles have been published in recent years: Yan et al. [30] conducted a retrospective multicohort diagnostic study, developing an AI cascading model for fully automated pulmonary TB diagnosis and triage using CT scans from 526 participants. The model achieved an overall accuracy of 81.1–91.1% in detecting six critical pulmonary imaging findings indicative of TB across independent datasets. Han et al. [31] developed a 3D-CNN model using chest CT to differentiate active pulmonary TB from community-acquired pneumonia, trained on 493 participants. The model achieved accuracies of 0.989 and 0.934 on internal and external test sets, respectively, with AUC values significantly surpassing those of two radiologists. However, as of now, there are currently no Conformité Européene (CE)-certified CAD products specifically designed for CT imaging. Existing CAD solutions are primarily developed for chest X-ray analysis and diagnosis. To evaluate our CXR TB screening AI model with localizer images of chest CT as a temporary bridge for underdeveloped CT CAD technology, results showed an optimal AI threshold of 0.111, with sensitivity of 0.612 and specificity of 0.717. While it falls short of WHO-defined effectiveness, it shows strong potential. Noted that among 105 pulmonary TB cases with both CXR and localizer images of the chest CT, the optimal threshold was 0.041, with a sensitivity of 0.952 and specificity of 0.873. Although the AUC may be overestimated due to inferred predictions for negatives, empirical evidence suggests the algorithm performs well. Using pulmonary infections instead of healthy controls may have underestimated its true performance.

After enhancing the performance for specific populations and settings, such as older individuals, those with a history of tuberculosis, and people living with HIV, we will consider integrating the AI tool into existing workflows. Ongoing monitoring of the AI system’s outcomes in practice, along with gathering user feedback, will help refine the system over time. However, fully replacing manual processes remains a long-term objective.

### Limitations

According to WHO’s Operational Handbook on TB (Module 2), if a program incorporates the use of CAD for automated interpretation of CXRs in screening or triage, it is crucial to calibrate the system to establish an appropriate threshold score. This calibration should be tailored to the specific setting and program, taking into account the spectrum of radiographic findings among the target population, both with and without TB disease [18]. In this study, the research population is relatively general, making it less challenging to identify an appropriate threshold score. However, in practical applications, it is necessary to consider calibrating the threshold score specifically for older individuals, children, individuals living with HIV, residents of remote areas, and different types of X-ray equipment etc. This requires further refinement and optimization in the future.

Additionally, TB patients and controls were not matched through stratified sampling based on age and gender, resulting in significant differences in the age and gender composition of the two groups. We cannot rule out the potential impact of these differences on the evaluation of the AI algorithm.

The algorithm was trained and validated on Chinese hospital data. External validation using imaging data from other populations was not available at this stage. To enhance translational value, a prospective multi-center study is planned to further evaluate the model’s performance across diverse populations and imaging environments, including comparisons between Chinese datasets and internationally trained models.

Another limitation is that the control group for CXR was made up of healthy individuals. In reality, it’s more useful to distinguish TB from other lung infections rather than from normal lungs. Also, we did not collect CT localizer images from healthy individuals, as they rarely undergo that in routine practice, making such data difficult to obtain. To make up for this, we used the CXR of healthy people to predict their CT scores. These predicted scores were used as the negative group for the CT test. Since they were not based on real CT images, the AUC result for localizer images of the chest CT may be overestimated. The findings based on CT localizer images should be interpreted as exploratory, given the inherent limitations in image quality and the potential bias in control selection.

Overall, our study represents an early but meaningful validation of the AI algorithm under real-world conditions. Although further external validation and demographic adjustment are warranted, the results provide useful preliminary evidence supporting its potential applicability in diverse clinical settings.

## Conclusions

The algorithm demonstrated good diagnostic performance on CXR samples in this study. Its application to CT localizer images provided exploratory insight, suggesting that some TB-related patterns can still be recognized in these low-resolution, non-diagnostic scans.

## Supporting information

S1 File Analysis code and data. Preprocessing scripts and model evaluation code are included.
(ZIP)
