# Supplementary material for: Validating the effectiveness of an AI algorithm for pulmonary tuberculosis screening using chest X-ray: Retrospective study and test accuracy with localizer images of the chest CT
Source: PLoS One. 2026 Feb 27;21(2):e0338810. doi: 10.1371/journal.pone.0338810 (PMC12948104; doi:10.1371/journal.pone.0338810)
Supplement: S1 File — Preprocessing scripts and model evaluation code are included. (ZIP) [file pone.0338810.s001.zip › CXR&Loc_code and data CHN original & ENG translation_anonymized/code and data English translated/Word version of the Rmd code.docx]

---

title: "AI Analysis of Pulmonary Tuberculosis"

author: "JiuFeng Medical"

---

```{r data, include=FALSE}

library(dplyr)

library(kableExtra)

library(showtext)

library(flextable)

library(officer)

library(caret)

library(ggplot2)

library(readxl)

library(tmcn)

library(lubridate)

library(DiagrammeR)

library(htmlwidgets)

library(webshot)

library(caret)

library(pROC)

library(ggvenn)

library(reportROC)

drdf0 <- readRDS("../data/drdf.rds")

locdf0 <- readRDS("../data/locdf.rds")

cttb1df0 <- readRDS("../data/cttb1df.rds")

cttb2df0 <- readRDS("../data/cttb2df.rds")

patdf0 <- readRDS("../data/patdf.rds")

drdf <- drdf0

drdf$aires <- NA

drdf$aires[drdf$tuberculosis > 0.085] <- "Active Pulmonary Tuberculosis"

drdf$aires[is.na(drdf$aires) & drdf$old_tuberculosis > 0.5] <- "Old Pulmonary Tuberculosis"

drdf$aires[is.na(drdf$aires) & apply(drdf[, c("cardiomegaly", "pleural_disease", "mass", "pneumothorax", "pneumonia")], 1, max) > 0.5] <- "Other Abnormalities"

drdf$aires[is.na(drdf$aires)] <- "No Abnormalities"

drdf$agegrp <- as.character(cut(drdf$age, breaks = c(-Inf, 20, 30, 40, 50, 60, 70, 80, 90, Inf)))

drdf <- merge(drdf, patdf0[, c("patno", "case", "lab")], all.x = TRUE)

locdf <- locdf0

locdf$aires <- NA

locdf$aires[locdf$tuberculosis > 0.35] <- "Active Pulmonary Tuberculosis"

locdf$aires[is.na(locdf$aires) & locdf$old_tuberculosis > 0.35] <- "Old Pulmonary Tuberculosis"

locdf$aires[is.na(locdf$aires) & apply(locdf[, c("cardiomegaly", "pleural_disease", "mass", "pneumothorax", "pneumonia")], 1, max) > 0.5] <- "Other Abnormalities"

locdf$aires[is.na(locdf$aires)] <- "No Abnormalities"

locdf$agegrp <- as.character(cut(locdf$age, breaks = c(-Inf, 20, 30, 40, 50, 60, 70, 80, 90, Inf)))

locdf <- merge(locdf, patdf0[, c("patno", "case", "lab")], all.x = TRUE)

ctdf <- cttb1df0

ctdf$aires <- NA

ctdf$aires[ctdf$tuberculosis > 0.35] <- "Active Pulmonary Tuberculosis"

#ctdf$aires[is.na(ctdf$aires) & ctdf$infection > 0.5] <- "Lung Infection"

ctdf$aires[is.na(ctdf$aires)] <- "No Abnormalities"

ctdf$agegrp <- as.character(cut(ctdf$age, breaks = c(-Inf, 20, 30, 40, 50, 60, 70, 80, 90, Inf)))

ctdf <- merge(ctdf, patdf0[, c("patno", "case", "lab")], all.x = TRUE)

\mainmatter

Data Overview

Data Cleaning

This study is based on the analysis of DR and CT images of pulmonary tuberculosis patients. The program scans the image folders, automatically identifies DICOM format files, and then runs JiuFeng Medical's AI algorithm for pulmonary tuberculosis to obtain the AI-predicted probability of active tuberculosis.

For cases with multiple images under the same patient ID, the record with the highest AI-predicted tuberculosis probability was selected. A very small number of cases with mixed patient IDs were excluded.

For CT images, 5mm-thick lung window plain scan images were extracted for AI analysis. Additionally, localizer images from CT scans were extracted and analyzed using the DR algorithm.

After data cleaning, a total of r nrow(drdf0) valid DR chest X-ray records, r nrow(cttb1df0) CT images, and r nrow(locdf0) CT localizer images were obtained. The relationships among the samples are shown in Figure@ref(fig:venn1).

{r

vennlist <- list("CXR" = drdf0$patno, "CT Localizer" = locdf0$patno, "CT" = cttb1df0$patno)

ggvenn(vennlist, auto_scale = FALSE)

Data Distribution

Among the r nrow(drdf) cases containing DR images, the average age was r round(mean(drdf$age), 2) years. There were r sum(drdf$sex %in% "Male") male patients (r round(sum(drdf$sex %in% "Male")/nrow(drdf)*100, 2)%) and r sum(drdf$sex %in% "Female") female patients (r round(sum(drdf$sex %in% "Female")/nrow(drdf)*100, 2)%).

Among the r nrow(ctdf) cases containing CT images, the average age was r round(mean(ctdf$age), 2) years. There were r sum(ctdf$sex %in% "Male") male patients (r round(sum(ctdf$sex %in% "Male")/nrow(ctdf)*100, 2)%) and r sum(ctdf$sex %in% "Female") female patients (r round(sum(ctdf$sex %in% "Female")/nrow(ctdf)*100, 2)%).

Among the r nrow(locdf) cases containing CT localizer images, the average age was r round(mean(locdf$age), 2) years. There were r sum(locdf$sex %in% "Male") male patients (r round(sum(locdf$sex %in% "Male")/nrow(locdf)*100, 2)%) and r sum(locdf$sex %in% "Female") female patients (r round(sum(locdf$sex %in% "Female")/nrow(locdf)*100, 2)%).

The age distribution of the cases is shown in Figure@ref(fig:distimg1), and the age quantiles are presented in Table@ref(tab:agetbl1).

{r

par(mar = c(4, 4, 1, 1), mfrow = c(1, 3))

hist(drdf$age, xlab = "Age (years)", ylab = "Frequency", main = "DR")

hist(ctdf$age, xlab = "Age (years)", ylab = "Frequency", main = "CT")

hist(locdf$age, xlab = "Age (years)", ylab = "Frequency", main = "CT Localizer")

{r

outdf <- data.frame(matrix(0, 4, 6))

outdf[[1]] <- c("DR", "CT", "CT Localizer", "Total")

outdf[1, 2:6] <- fivenum(drdf$age, na.rm = TRUE)

outdf[2, 2:6] <- fivenum(ctdf$age, na.rm = TRUE)

outdf[3, 2:6] <- fivenum(locdf$age, na.rm = TRUE)

outdf[4, 2:6] <- fivenum(c(drdf$age, ctdf$age, locdf$age), na.rm = TRUE)

colnames(outdf) <- c("Sample Type", "Minimum", "Lower Quartile", "Median", "Upper Quartile", "Maximum")

regulartable(outdf) %>%

theme_vanilla() %>%

fontsize(size = 8, part = "header") %>%

fontsize(size = 7, part = "body") %>%

vline(i = NULL, j = c(1), border = fp_border(color="black"), part = "all") %>%

hline(i = nrow(outdf)-1, j = NULL, border = fp_border(width=1.5, color=gray(0.4)), part = "body") %>%

align(i = NULL, j = 1, align = "left", part = "all") %>%

align(i = NULL, j = 2:ncol(outdf), align = "center", part = "all") %>%

width(j = 1, width = 0.6) %>%

width(j = 2:ncol(outdf), width = 0.7) %>%

set_caption("Age Quantiles")

DR Image Analysis

Prediction Accuracy Analysis Based on the DR Model

In this study, DR chest X-rays of healthy individuals were used as the control group, with a total of r sum(drdf$label %in% "normal") samples. The average age of the control group was r round(mean(drdf$age[drdf$label %in% "normal"]), 2) years, with r sum(drdf$label %in% "normal" & drdf$sex %in% "Male") male patients (r round(sum(drdf$label %in% "normal" & drdf$sex %in% "Male")/sum(drdf$label %in% "normal")*100, 2)%) and r sum(drdf$label %in% "normal" & drdf$sex %in% "Female") female patients (r round(sum(drdf$label %in% "normal" & drdf$sex %in% "Female")/sum(drdf$label %in% "normal")*100, 2)%).

The average age of positive samples was r round(mean(drdf$age[drdf$label %in% "tb"]), 2) years, with r sum(drdf$label %in% "tb" & drdf$sex %in% "Male") male patients (r round(sum(drdf$label %in% "tb" & drdf$sex %in% "Male")/sum(drdf$label %in% "tb")*100, 2)%) and r sum(drdf$label %in% "tb" & drdf$sex %in% "Female") female patients (r round(sum(drdf$label %in% "tb" & drdf$sex %in% "Female")/sum(drdf$label %in% "tb")*100, 2)%).

The age distribution of different sample groups is shown in Figure@ref(fig:distsamp1), and the age quantiles are presented in Table@ref(tab:agesamp1).

{r

par(mar = c(4, 4, 1, 1), mfrow = c(1, 2))

hist(drdf$age[drdf$label %in% "normal"], xlab = "Age (years)", ylab = "Frequency", main = "Negative Samples")

hist(drdf$age[drdf$label %in% "tb"], xlab = "Age (years)", ylab = "Frequency", main = "Positive Samples")

{r

outdf <- data.frame(matrix(0, 3, 6))

outdf[[1]] <- c("Negative", "Positive", "Total")

outdf[2, 2:6] <- fivenum(drdf$age[drdf$label %in% "normal"], na.rm = TRUE)

outdf[1, 2:6] <- fivenum(drdf$age[drdf$label %in% "tb"], na.rm = TRUE)

outdf[3, 2:6] <- fivenum(drdf$age, na.rm = TRUE)

colnames(outdf) <- c("Sample Type", "Minimum", "Lower Quartile", "Median", "Upper Quartile", "Maximum")

regulartable(outdf) %>%

theme_vanilla() %>%

fontsize(size = 8, part = "header") %>%

fontsize(size = 7, part = "body") %>%

vline(i = NULL, j = c(1), border = fp_border(color="black"), part = "all") %>%

hline(i = nrow(outdf)-1, j = NULL, border = fp_border(width=1.5, color=gray(0.4)), part = "body") %>%

align(i = NULL, j = 1, align = "left", part = "all") %>%

align(i = NULL, j = 2:ncol(outdf), align = "center", part = "all") %>%

width(j = 1, width = 0.6) %>%

width(j = 2:ncol(outdf), width = 0.7) %>%

set_caption("Age Quantiles")

drdf$Y <- 1

drdf$Y[drdf$label %in% "normal"] <- 0

roc1 <- roc(drdf$Y, drdf$tuberculosis)

Using the diagnostic results from this study and the test set as the gold standard, the AI algorithm based on DR was used for prediction. The ROC curve of AI prediction results is shown in Figure@ref(fig:roc1). On the confirmed case dataset, the optimal threshold for AI was r round(coords(roc1, "best")[["threshold"]], 3), with a sensitivity of r round(coords(roc1, "best")[["sensitivity"]], 3) and a specificity of r round(coords(roc1, "best")[["specificity"]], 3).

{r

plot(roc1, print.auc=TRUE, auc.polygon=TRUE, grid=c(0.1, 0.2),

grid.col=c("green", "red"), max.auc.polygon=TRUE,

auc.polygon.col="lightblue", print.thres=TRUE)

Based on different thresholds, various accuracy metrics and their 95% confidence intervals were calculated, as shown in Table@ref(tab:conftbl1).

{r

drdf$tb1 <- 0

drdf$tb1[drdf$tuberculosis > 0.35] <- 1

drdf$tb2 <- 0

drdf$tb2[drdf$tuberculosis > coords(roc1, "best")[["threshold"]]] <- 1

rocrpt0 <- reportROC(gold = as.factor(drdf$Y), predictor = drdf$tuberculosis, important = "se", plot = FALSE)

rocrpt1 <- reportROC(gold = as.factor(drdf$Y), predictor.binary = as.factor(drdf$tb1), important = "se", plot = FALSE)

rocrpt2 <- reportROC(gold = as.factor(drdf$Y), predictor.binary = as.factor(drdf$tb2), important = "se", plot = FALSE)

outdf <- data.frame(matrix(0, 2, 6))

outdf[[1]] <- c(0.35, round(coords(roc1, "best")[["threshold"]], 3))

outdf[1, 2] <- paste0(rocrpt0[["ACC"]], "\n(", rocrpt0[["ACC.low"]], " - ", rocrpt0[["ACC.up"]], ")")

outdf[1, 3] <- paste0(rocrpt1[["SEN"]], "\n(", rocrpt1[["SEN.low"]], " - ", rocrpt1[["SEN.up"]], ")")

outdf[1, 4] <- paste0(rocrpt1[["SPE"]], "\n(", rocrpt1[["SPE.low"]], " - ", rocrpt1[["SPE.up"]], ")")

outdf[1, 5] <- paste0(rocrpt0[["AUC"]], "\n(", rocrpt0[["AUC.low"]], " - ", rocrpt0[["AUC.up"]], ")")

outdf[1, 6] <- paste0(rocrpt0[["KAPPA"]], "\n(", rocrpt0[["KAPPA.low"]], " - ", rocrpt0[["KAPPA.up"]], ")")

outdf[2, 2] <- paste0(rocrpt0[["ACC"]], "\n(", rocrpt0[["ACC.low"]], " - ", rocrpt0[["ACC.up"]], ")")

outdf[2, 3] <- paste0(rocrpt2[["SEN"]], "\n(", rocrpt2[["SEN.low"]], " - ", rocrpt2[["SEN.up"]], ")")

outdf[2, 4] <- paste0(rocrpt2[["SPE"]], "\n(", rocrpt2[["SPE.low"]], " - ", rocrpt2[["SPE.up"]], ")")

outdf[2, 5] <- paste0(rocrpt0[["AUC"]], "\n(", rocrpt0[["AUC.low"]], " - ", rocrpt0[["AUC.up"]], ")")

outdf[2, 6] <- paste0(rocrpt0[["KAPPA"]], "\n(", rocrpt0[["KAPPA.low"]], " - ", rocrpt0[["KAPPA.up"]], ")")

colnames(outdf) <- c("Threshold", "Accuracy", "Sensitivity", "Specificity", "AUC", "Kappa Value")

regulartable(outdf) %>%

theme_vanilla() %>%

fontsize(size = 8, part = "header") %>%

fontsize(size = 7, part = "body") %>%

vline(i = NULL, j = c(1), border = fp_border(color="black"), part = "all") %>%

align(i = NULL, j = 1, align = "left", part = "all") %>%

align(i = NULL, j = 2:ncol(outdf), align = "center", part = "all") %>%

width(j = 1, width = 0.7) %>%

width(j = 2:ncol(outdf), width = 0.9) %>%

set_caption("Accuracy Metrics of AI Evaluation Results")

Analysis of Prediction Results for Positive Cases

This study focuses on the prediction accuracy of active pulmonary tuberculosis. In addition to outputting the predicted probability of active tuberculosis, the AI also outputs predictions for old tuberculosis, pneumonia, pneumothorax, nodules/masses, pleural lesions, cardiomegaly, and other abnormalities. We categorized the prediction results into four groups: active pulmonary tuberculosis, old pulmonary tuberculosis, other abnormalities, and no abnormality. The prediction proportions for different diagnostic results are shown in Table@ref(tab:disttbl1).

{r

outdf0 <- summarise(group_by(drdf[drdf$label %in% "tb", ], case, lab), num = length(patno), tb = sum(aires %in% "Active Pulmonary Tuberculosis"), tbrate = 0, oldtb = sum(aires %in% "Old Pulmonary Tuberculosis"), other = sum(aires %in% "Other Abnormalities"), norm = sum(aires %in% "No Abnormalities"))

outdf <- rbind(as.data.frame(lapply(outdf0[outdf0$case %in% "Confirmed Cases", ], FUN = function(X) ifelse(!inherits(X, "character"), sum(X), ifelse(grepl("Case", X[1]), X[1], "")))),

outdf0[outdf0$case %in% "Confirmed Cases", ],

as.data.frame(lapply(outdf0[outdf0$case %in% "Clinically Diagnosed Cases", ], FUN = function(X) ifelse(!inherits(X, "character"), sum(X), ifelse(grepl("Case", X[1]), X[1], "")))),

outdf0[outdf0$case %in% "Clinically Diagnosed Cases", ],

as.data.frame(lapply(outdf0[outdf0$case %in% "Suspected Cases", ], FUN = function(X) ifelse(!inherits(X, "character"), sum(X), ifelse(grepl("Case", X[1]), X[1], "")))),

outdf0[outdf0$case %in% "Suspected Cases", ],

as.data.frame(lapply(outdf0, FUN = function(X) ifelse(!inherits(X, "character"), sum(X), ""))))

outdf$case[nzchar(outdf$lab)] <- ""

outdf$case[nrow(outdf)] <- "Total"

outdf$tbrate <- paste0(format(outdf$tb / outdf$num * 100, digits = 1, nsmall = 1), "%")

outdf$abn <- paste0(format(100 - outdf$norm / outdf$num * 100, digits = 1, nsmall = 1), "%")

colnames(outdf) <- c("Case Category", "Test Result", "Number of Cases", "Active Pulmonary Tuberculosis", "Tuberculosis Positive Rate", "Old Pulmonary Tuberculosis", "Other Abnormalities", "No Abnormalities", "Abnormality Rate")

regulartable(outdf) %>%

theme_vanilla() %>%

fontsize(size = 8, part = "header") %>%

fontsize(size = 7, part = "body") %>%

hline(i = c(sum(outdf0$case %in% "Confirmed Cases") + 1, sum(outdf0$case %in% c("Confirmed Cases", "Clinically Diagnosed Cases")) + 2, nrow(outdf)-1), j = NULL, border = fp_border(width=1.5, color = gray(0.4)), part = "body") %>%

align(i = NULL, j = 1, align = "left", part = "all") %>%

align(i = NULL, j = 2:ncol(outdf), align = "center", part = "all") %>%

width(j = 1, width = 0.8) %>%

width(j = 2, width = 1.1) %>%

width(j = c(3:6, 9), width = 0.5) %>%

width(j = 7:8, width = 0.4) %>%

set_caption("Accuracy by Category")

CT Image Analysis

Prediction Accuracy Analysis Based on the CT Model

In this study, CT images of lung infections were used as the control group, with a total of r sum(ctdf$label %in% "infection") samples. The average age of the control group was r round(mean(ctdf$age[ctdf$label %in% "infection"]), 2) years, with r sum(ctdf$label %in% "infection" & ctdf$sex %in% "Male") male patients (r round(sum(ctdf$label %in% "infection" & ctdf$sex %in% "Male")/sum(ctdf$label %in% "infection")*100, 2)%) and r sum(ctdf$label %in% "infection" & ctdf$sex %in% "Female") female patients (r round(sum(ctdf$label %in% "infection" & ctdf$sex %in% "Female")/sum(ctdf$label %in% "infection")*100, 2)%).

The average age of positive samples was r round(mean(ctdf$age[ctdf$label %in% "tb"]), 2) years, with r sum(ctdf$label %in% "tb" & ctdf$sex %in% "Male") male patients (r round(sum(ctdf$label %in% "tb" & ctdf$sex %in% "Male")/sum(ctdf$label %in% "tb")*100, 2)%) and r sum(ctdf$label %in% "tb" & ctdf$sex %in% "Female") female patients (r round(sum(ctdf$label %in% "tb" & ctdf$sex %in% "Female")/sum(ctdf$label %in% "tb")*100, 2)%).

The age distribution of different sample groups is shown in Figure@ref(fig:distsamp2), and the age quantiles are presented in Table@ref(tab:agesamp2).

{r

par(mar = c(4, 4, 1, 1), mfrow = c(1, 2))

hist(ctdf$age[ctdf$label %in% "infection"], xlab = "Age (years)", ylab = "Frequency", main = "Control Samples")

hist(ctdf$age[ctdf$label %in% "tb"], xlab = "Age (years)", ylab = "Frequency", main = "Positive Samples")

{r

outdf <- data.frame(matrix(0, 3, 6))

outdf[[1]] <- c("Control", "Positive", "Total")

outdf[2, 2:6] <- fivenum(ctdf$age[ctdf$label %in% "infection"], na.rm = TRUE)

outdf[1, 2:6] <- fivenum(ctdf$age[ctdf$label %in% "tb"], na.rm = TRUE)

outdf[3, 2:6] <- fivenum(ctdf$age, na.rm = TRUE)

colnames(outdf) <- c("Sample Type", "Minimum", "Lower Quartile", "Median", "Upper Quartile", "Maximum")

regulartable(outdf) %>%

theme_vanilla() %>%

fontsize(size = 8, part = "header") %>%

fontsize(size = 7, part = "body") %>%

vline(i = NULL, j = c(1), border = fp_border(color="black"), part = "all") %>%

hline(i = nrow(outdf)-1, j = NULL, border = fp_border(width=1.5, color=gray(0.4)), part = "body") %>%

align(i = NULL, j = 1, align = "left", part = "all") %>%

align(i = NULL, j = 2:ncol(outdf), align = "center", part = "all") %>%

width(j = 1, width = 0.6) %>%

width(j = 2:ncol(outdf), width = 0.7) %>%

set_caption("Age Quantiles")

ctdf$Y <- 1

ctdf$Y[ctdf$label %in% "infection"] <- 0

roc1 <- roc(ctdf$Y, ctdf$tuberculosis)

Using the diagnostic results from this study and the test set as the gold standard, the AI algorithm based on CT was used for prediction. The ROC curve of AI prediction results is shown in Figure@ref(fig:roc2). On the confirmed case dataset, the optimal threshold for AI was r round(coords(roc1, "best")[["threshold"]], 3), with a sensitivity of r round(coords(roc1, "best")[["sensitivity"]], 3) and a specificity of r round(coords(roc1, "best")[["specificity"]], 3).

{r

plot(roc1, print.auc=TRUE, auc.polygon=TRUE, grid=c(0.1, 0.2),

grid.col=c("green", "red"), max.auc.polygon=TRUE,

auc.polygon.col="lightblue", print.thres=TRUE)

Based on different thresholds, various accuracy metrics and their 95% confidence intervals were calculated, as shown in Table@ref(tab:conftbl2).

{r

ctdf$tb1 <- 0

ctdf$tb1[ctdf$tuberculosis > 0.5] <- 1

ctdf$tb2 <- 0

ctdf$tb2[ctdf$tuberculosis > coords(roc1, "best")[["threshold"]]] <- 1

rocrpt0 <- reportROC(gold = as.factor(ctdf$Y), predictor = ctdf$tuberculosis, important = "se", plot = FALSE)

rocrpt1 <- reportROC(gold = as.factor(ctdf$Y), predictor.binary = as.factor(ctdf$tb1), important = "se", plot = FALSE)

rocrpt2 <- reportROC(gold = as.factor(ctdf$Y), predictor.binary = as.factor(ctdf$tb2), important = "se", plot = FALSE)

outdf <- data.frame(matrix(0, 2, 6))

outdf[[1]] <- c(0.5, round(coords(roc1, "best")[["threshold"]], 3))

outdf[1, 2] <- paste0(rocrpt0[["ACC"]], "\n(", rocrpt0[["ACC.low"]], " - ", rocrpt0[["ACC.up"]], ")")

outdf[1, 3] <- paste0(rocrpt1[["SEN"]], "\n(", rocrpt1[["SEN.low"]], " - ", rocrpt1[["SEN.up"]], ")")

outdf[1, 4] <- paste0(rocrpt1[["SPE"]], "\n(", rocrpt1[["SPE.low"]], " - ", rocrpt1[["SPE.up"]], ")")

outdf[1, 5] <- paste0(rocrpt0[["AUC"]], "\n(", rocrpt0[["AUC.low"]], " - ", rocrpt0[["AUC.up"]], ")")

outdf[1, 6] <- paste0(rocrpt0[["KAPPA"]], "\n(", rocrpt0[["KAPPA.low"]], " - ", rocrpt0[["KAPPA.up"]], ")")

outdf[2, 2] <- paste0(rocrpt0[["ACC"]], "\n(", rocrpt0[["ACC.low"]], " - ", rocrpt0[["ACC.up"]], ")")

outdf[2, 3] <- paste0(rocrpt2[["SEN"]], "\n(", rocrpt2[["SEN.low"]], " - ", rocrpt2[["SEN.up"]], ")")

outdf[2, 4] <- paste0(rocrpt2[["SPE"]], "\n(", rocrpt2[["SPE.low"]], " - ", rocrpt2[["SPE.up"]], ")")

outdf[2, 5] <- paste0(rocrpt0[["AUC"]], "\n(", rocrpt0[["AUC.low"]], " - ", rocrpt0[["AUC.up"]], ")")

outdf[2, 6] <- paste0(rocrpt0[["KAPPA"]], "\n(", rocrpt0[["KAPPA.low"]], " - ", rocrpt0[["KAPPA.up"]], ")")

colnames(outdf) <- c("Threshold", "Accuracy", "Sensitivity", "Specificity", "AUC", "Kappa Value")

regulartable(outdf) %>%

theme_vanilla() %>%

fontsize(size = 8, part = "header") %>%

fontsize(size = 7, part = "body") %>%

vline(i = NULL, j = c(1), border = fp_border(color="black"), part = "all") %>%

align(i = NULL, j = 1, align = "left", part = "all") %>%

align(i = NULL, j = 2:ncol(outdf), align = "center", part = "all") %>%

width(j = 1, width = 0.7) %>%

width(j = 2:ncol(outdf), width = 0.9) %>%

set_caption("Accuracy Metrics of AI Evaluation Results")

Prediction Accuracy Analysis Based on the Optimized CT Model

{r

ctdf <- cttb2df0

ctdf$aires <- NA

ctdf$aires[ctdf$tuberculosis > 0.938] <- "Active Pulmonary Tuberculosis"

ctdf$aires[is.na(ctdf$aires)] <- "Other"

ctdf$agegrp <- as.character(cut(ctdf$age, breaks = c(-Inf, 20, 30, 40, 50, 60, 70, 80, 90, Inf)))

ctdf <- merge(ctdf, patdf0[, c("patno", "case", "lab")], all.x = TRUE)

ctdf$Y <- 1

ctdf$Y[ctdf$label %in% "infection"] <- 0

roc1 <- roc(ctdf$Y, ctdf$tuberculosis)

Using the optimized AI algorithm for prediction and evaluating with 5-fold cross-validation, the ROC curve of AI prediction results is shown in Figure@ref(fig:roc3). On the confirmed case dataset, the optimal threshold for AI was r round(coords(roc1, "best")[["threshold"]], 3), with a sensitivity of r round(coords(roc1, "best")[["sensitivity"]], 3) and a specificity of r round(coords(roc1, "best")[["specificity"]], 3).

{r

plot(roc1, print.auc=TRUE, auc.polygon=TRUE, grid=c(0.1, 0.2),

grid.col=c("green", "red"), max.auc.polygon=TRUE,

auc.polygon.col="lightblue", print.thres=TRUE)

Based on different thresholds, various accuracy metrics and their 95% confidence intervals were calculated, as shown in Table@ref(tab:conftbl3).

{r

ctdf$tb1 <- 0

ctdf$tb1[ctdf$tuberculosis > 0.5] <- 1

ctdf$tb2 <- 0

ctdf$tb2[ctdf$tuberculosis > coords(roc1, "best")[["threshold"]]] <- 1

rocrpt0 <- reportROC(gold = as.factor(ctdf$Y), predictor = ctdf$tuberculosis, important = "se", plot = FALSE)

rocrpt1 <- reportROC(gold = as.factor(ctdf$Y), predictor.binary = as.factor(ctdf$tb1), important = "se", plot = FALSE)

rocrpt2 <- reportROC(gold = as.factor(ctdf$Y), predictor.binary = as.factor(ctdf$tb2), important = "se", plot = FALSE)

outdf <- data.frame(matrix(0, 2, 6))

outdf[[1]] <- c(0.5, round(coords(roc1, "best")[["threshold"]], 3))

outdf[1, 2] <- paste0(rocrpt0[["ACC"]], "\n(", rocrpt0[["ACC.low"]], " - ", rocrpt0[["ACC.up"]], ")")

outdf[1, 3] <- paste0(rocrpt1[["SEN"]], "\n(", rocrpt1[["SEN.low"]], " - ", rocrpt1[["SEN.up"]], ")")

outdf[1, 4] <- paste0(rocrpt1[["SPE"]], "\n(", rocrpt1[["SPE.low"]], " - ", rocrpt1[["SPE.up"]], ")")

outdf[1, 5] <- paste0(rocrpt0[["AUC"]], "\n(", rocrpt0[["AUC.low"]], " - ", rocrpt0[["AUC.up"]], ")")

outdf[1, 6] <- paste0(rocrpt0[["KAPPA"]], "\n(", rocrpt0[["KAPPA.low"]], " - ", rocrpt0[["KAPPA.up"]], ")")

outdf[2, 2] <- paste0(rocrpt0[["ACC"]], "\n(", rocrpt0[["ACC.low"]], " - ", rocrpt0[["ACC.up"]], ")")

outdf[2, 3] <- paste0(rocrpt2[["SEN"]], "\n(", rocrpt2[["SEN.low"]], " - ", rocrpt2[["SEN.up"]], ")")

outdf[2, 4] <- paste0(rocrpt2[["SPE"]], "\n(", rocrpt2[["SPE.low"]], " - ", rocrpt2[["SPE.up"]], ")")

outdf[2, 5] <- paste0(rocrpt0[["AUC"]], "\n(", rocrpt0[["AUC.low"]], " - ", rocrpt0[["AUC.up"]], ")")

outdf[2, 6] <- paste0(rocrpt0[["KAPPA"]], "\n(", rocrpt0[["KAPPA.low"]], " - ", rocrpt0[["KAPPA.up"]], ")")

colnames(outdf) <- c("Threshold", "Accuracy", "Sensitivity", "Specificity", "AUC", "Kappa Value")

regulartable(outdf) %>%

theme_vanilla() %>%

fontsize(size = 8, part = "header") %>%

fontsize(size = 7, part = "body") %>%

vline(i = NULL, j = c(1), border = fp_border(color="black"), part = "all") %>%

align(i = NULL, j = 1, align = "left", part = "all") %>%

align(i = NULL, j = 2:ncol(outdf), align = "center", part = "all") %>%

width(j = 1, width = 0.7) %>%

width(j = 2:ncol(outdf), width = 0.9) %>%

set_caption("Accuracy Metrics of AI Evaluation Results")

Analysis of Prediction Results for Positive Cases

This study focuses on the prediction accuracy of active pulmonary tuberculosis and its differential diagnosis from lung infections. The prediction accuracy for different categories is shown in Table@ref(tab:disttbl2).

{r

outdf0 <- summarise(group_by(ctdf[ctdf$label %in% "tb", ], case, lab), num = length(patno), tb = sum(aires %in% "Active Pulmonary Tuberculosis"), tbrate = 0)

outdf <- rbind(as.data.frame(lapply(outdf0[outdf0$case %in% "Confirmed Cases", ], FUN = function(X) ifelse(!inherits(X, "character"), sum(X), ifelse(grepl("Case", X[1]), X[1], "")))),

outdf0[outdf0$case %in% "Confirmed Cases", ],

as.data.frame(lapply(outdf0[outdf0$case %in% "Clinically Diagnosed Cases", ], FUN = function(X) ifelse(!inherits(X, "character"), sum(X), ifelse(grepl("Case", X[1]), X[1], "")))),

outdf0[outdf0$case %in% "Clinically Diagnosed Cases", ],

as.data.frame(lapply(outdf0[outdf0$case %in% "Suspected Cases", ], FUN = function(X) ifelse(!inherits(X, "character"), sum(X), ifelse(grepl("Case", X[1]), X[1], "")))),

outdf0[outdf0$case %in% "Suspected Cases", ],

as.data.frame(lapply(outdf0, FUN = function(X) ifelse(!inherits(X, "character"), sum(X), ""))))

outdf$case[nzchar(outdf$lab)] <- ""

outdf$case[nrow(outdf)] <- "Total"

outdf$tbrate <- paste0(format(outdf$tb / outdf$num * 100, digits = 1, nsmall = 1), "%")

colnames(outdf) <- c("Case Category", "Test Result", "Number of Cases", "Active Pulmonary Tuberculosis", "Tuberculosis Positive Rate")

regulartable(outdf) %>%

theme_vanilla() %>%

fontsize(size = 8, part = "header") %>%

fontsize(size = 7, part = "body") %>%

hline(i = c(sum(outdf0$case %in% "Confirmed Cases") + 1, sum(outdf0$case %in% c("Confirmed Cases", "Clinically Diagnosed Cases")) + 2, nrow(outdf)-1), j = NULL, border = fp_border(width=1.5, color = gray(0.4)), part = "body") %>%

align(i = NULL, j = 1, align = "left", part = "all") %>%

align(i = NULL, j = 2:ncol(outdf), align = "center", part = "all") %>%

width(j = 1, width = 0.8) %>%

width(j = 2, width = 1.1) %>%

width(j = c(3:5), width = 0.5) %>%

set_caption("Accuracy by Category")

CT Localizer Image Analysis

Prediction Accuracy Analysis Based on the DR Model

In this study, CT localizer images of lung infections were used as the control group, with a total of r sum(locdf$label %in% "infection") samples. The average age of the control group was r round(mean(locdf$age[locdf$label %in% "infection"]), 2) years, with r sum(locdf$label %in% "infection" & locdf$sex %in% "Male") male patients (r round(sum(locdf$label %in% "infection" & locdf$sex %in% "Male")/sum(locdf$label %in% "infection")*100, 2)%) and r sum(locdf$label %in% "infection" & locdf$sex %in% "Female") female patients (r round(sum(locdf$label %in% "infection" & locdf$sex %in% "Female")/sum(locdf$label %in% "infection")*100, 2)%).

The average age of positive samples was r round(mean(locdf$age[locdf$label %in% "tb"]), 2) years, with r sum(locdf$label %in% "tb" & locdf$sex %in% "Male") male patients (r round(sum(locdf$label %in% "tb" & locdf$sex %in% "Male")/sum(locdf$label %in% "tb")*100, 2)%) and r sum(locdf$label %in% "tb" & locdf$sex %in% "Female") female patients (r round(sum(locdf$label %in% "tb" & locdf$sex %in% "Female")/sum(locdf$label %in% "tb")*100, 2)%).

The age distribution of different sample groups is shown in Figure@ref(fig:distsamp3), and the age quantiles are presented in Table@ref(tab:agesamp3).

{r

par(mar = c(4, 4, 1, 1), mfrow = c(1, 2))

hist(locdf$age[locdf$label %in% "infection"], xlab = "Age (years)", ylab = "Frequency", main = "Negative Samples")

hist(locdf$age[locdf$label %in% "tb"], xlab = "Age (years)", ylab = "Frequency", main = "Positive Samples")

{r

outdf <- data.frame(matrix(0, 3, 6))

outdf[[1]] <- c("Negative", "Positive", "Total")

outdf[2, 2:6] <- fivenum(locdf$age[locdf$label %in% "infection"], na.rm = TRUE)

outdf[1, 2:6] <- fivenum(locdf$age[locdf$label %in% "tb"], na.rm = TRUE)

outdf[3, 2:6] <- fivenum(locdf$age, na.rm = TRUE)

colnames(outdf) <- c("Sample Type", "Minimum", "Lower Quartile", "Median", "Upper Quartile", "Maximum")

regulartable(outdf) %>%

theme_vanilla() %>%

fontsize(size = 8, part = "header") %>%

fontsize(size = 7, part = "body") %>%

vline(i = NULL, j = c(1), border = fp_border(color="black"), part = "all") %>%

hline(i = nrow(outdf)-1, j = NULL, border = fp_border(width=1.5, color=gray(0.4)), part = "body") %>%

align(i = NULL, j = 1, align = "left", part = "all") %>%

align(i = NULL, j = 2:ncol(outdf), align = "center", part = "all") %>%

width(j = 1, width = 0.6) %>%

width(j = 2:ncol(outdf), width = 0.7) %>%

set_caption("Age Quantiles")

locdf$Y <- 1

locdf$Y[locdf$label %in% "infection"] <- 0

roc1 <- roc(locdf$Y, locdf$tuberculosis)

Using the diagnostic results from this study and the test set as the gold standard, the AI algorithm based on DR was used for prediction. The ROC curve of AI prediction results is shown in Figure@ref(fig:roc4). On the confirmed case dataset, the optimal threshold for AI was r round(coords(roc1, "best")[["threshold"]], 3), with a sensitivity of r round(coords(roc1, "best")[["sensitivity"]], 3) and a specificity of r round(coords(roc1, "best")[["specificity"]], 3).

{r

plot(roc1, print.auc=TRUE, auc.polygon=TRUE, grid=c(0.1, 0.2),

grid.col=c("green", "red"), max.auc.polygon=TRUE,

auc.polygon.col="lightblue", print.thres=TRUE)

Based on different thresholds, various accuracy metrics and their 95% confidence intervals were calculated, as shown in Table@ref(tab:conftbl4).

{r

locdf$tb1 <- 0

locdf$tb1[locdf$tuberculosis > 0.35] <- 1

locdf$tb2 <- 0

locdf$tb2[locdf$tuberculosis > coords(roc1, "best")[["threshold"]]] <- 1

rocrpt0 <- reportROC(gold = as.factor(locdf$Y), predictor = locdf$tuberculosis, important = "se", plot = FALSE)

rocrpt1 <- reportROC(gold = as.factor(locdf$Y), predictor.binary = as.factor(locdf$tb1), important = "se", plot = FALSE)

rocrpt2 <- reportROC(gold = as.factor(locdf$Y), predictor.binary = as.factor(locdf$tb2), important = "se", plot = FALSE)

outdf <- data.frame(matrix(0, 2, 6))

outdf[[1]] <- c(0.35, round(coords(roc1, "best")[["threshold"]], 3))

outdf[1, 2] <- paste0(rocrpt0[["ACC"]], "\n(", rocrpt0[["ACC.low"]], " - ", rocrpt0[["ACC.up"]], ")")

outdf[1, 3] <- paste0(rocrpt1[["SEN"]], "\n(", rocrpt1[["SEN.low"]], " - ", rocrpt1[["SEN.up"]], ")")

outdf[1, 4] <- paste0(rocrpt1[["SPE"]], "\n(", rocrpt1[["SPE.low"]], " - ", rocrpt1[["SPE.up"]], ")")

outdf[1, 5] <- paste0(rocrpt0[["AUC"]], "\n(", rocrpt0[["AUC.low"]], " - ", rocrpt0[["AUC.up"]], ")")

outdf[1, 6] <- paste0(rocrpt0[["KAPPA"]], "\n(", rocrpt0[["KAPPA.low"]], " - ", rocrpt0[["KAPPA.up"]], ")")

outdf[2, 2] <- paste0(rocrpt0[["ACC"]], "\n(", rocrpt0[["ACC.low"]], " - ", rocrpt0[["ACC.up"]], ")")

outdf[2, 3] <- paste0(rocrpt2[["SEN"]], "\n(", rocrpt2[["SEN.low"]], " - ", rocrpt2[["SEN.up"]], ")")

outdf[2, 4] <- paste0(rocrpt2[["SPE"]], "\n(", rocrpt2[["SPE.low"]], " - ", rocrpt2[["SPE.up"]], ")")

outdf[2, 5] <- paste0(rocrpt0[["AUC"]], "\n(", rocrpt0[["AUC.low"]], " - ", rocrpt0[["AUC.up"]], ")")

outdf[2, 6] <- paste0(rocrpt0[["KAPPA"]], "\n(", rocrpt0[["KAPPA.low"]], " - ", rocrpt0[["KAPPA.up"]], ")")

colnames(outdf) <- c("Threshold", "Accuracy", "Sensitivity", "Specificity", "AUC", "Kappa Value")

regulartable(outdf) %>%

theme_vanilla() %>%

fontsize(size = 8, part = "header") %>%

fontsize(size = 7, part = "body") %>%

vline(i = NULL, j = c(1), border = fp_border(color="black"), part = "all") %>%

align(i = NULL, j = 1, align = "left", part = "all") %>%

align(i = NULL, j = 2:ncol(outdf), align = "center", part = "all") %>%

width(j = 1, width = 0.7) %>%

width(j = 2:ncol(outdf), width = 0.9) %>%

set_caption("Accuracy Metrics of AI Evaluation Results")

Analysis of Prediction Results for Positive Cases

This study focuses on the prediction accuracy of active pulmonary tuberculosis. In addition to outputting the predicted probability of active tuberculosis, the AI also outputs predictions for old tuberculosis, pneumonia, pneumothorax, nodules/masses, pleural lesions, cardiomegaly, and other abnormalities. We categorized the prediction results into four groups: active pulmonary tuberculosis, old pulmonary tuberculosis, other abnormalities, and no abnormality. The prediction proportions for different diagnostic results are shown in Table@ref(tab:disttbl3).

{r

outdf0 <- summarise(group_by(locdf[locdf$label %in% "tb", ], case, lab), num = length(patno), tb = sum(aires %in% "Active Pulmonary Tuberculosis"), tbrate = 0, oldtb = sum(aires %in% "Old Pulmonary Tuberculosis"), other = sum(aires %in% "Other Abnormalities"), norm = sum(aires %in% "No Abnormalities"))

outdf <- rbind(as.data.frame(lapply(outdf0[outdf0$case %in% "Confirmed Cases", ], FUN = function(X) ifelse(!inherits(X, "character"), sum(X), ifelse(grepl("Case", X[1]), X[1], "")))),

outdf0[outdf0$case %in% "Confirmed Cases", ],

as.data.frame(lapply(outdf0[outdf0$case %in% "Clinically Diagnosed Cases", ], FUN = function(X) ifelse(!inherits(X, "character"), sum(X), ifelse(grepl("Case", X[1]), X[1], "")))),

outdf0[outdf0$case %in% "Clinically Diagnosed Cases", ],

as.data.frame(lapply(outdf0[outdf0$case %in% "Suspected Cases", ], FUN = function(X) ifelse(!inherits(X, "character"), sum(X), ifelse(grepl("Case", X[1]), X[1], "")))),

outdf0[outdf0$case %in% "Suspected Cases", ],

as.data.frame(lapply(outdf0, FUN = function(X) ifelse(!inherits(X, "character"), sum(X), ""))))

outdf$case[nzchar(outdf$lab)] <- ""

outdf$case[nrow(outdf)] <- "Total"

outdf$tbrate <- paste0(format(outdf$tb / outdf$num * 100, digits = 1, nsmall = 1), "%")

outdf$abn <- paste0(format(100 - outdf$norm / outdf$num * 100, digits = 1, nsmall = 1), "%")

colnames(outdf) <- c("Case Category", "Test Result", "Number of Cases", "Active Pulmonary Tuberculosis", "Tuberculosis Positive Rate", "Old Pulmonary Tuberculosis", "Other Abnormalities", "No Abnormalities", "Abnormality Rate")

regulartable(outdf) %>%

theme_vanilla() %>%

fontsize(size = 8, part = "header") %>%

fontsize(size = 7, part = "body") %>%

hline(i = c(sum(outdf0$case %in% "Confirmed Cases") + 1, sum(outdf0$case %in% c("Confirmed Cases", "Clinically Diagnosed Cases")) + 2, nrow(outdf)-1), j = NULL, border = fp_border(width=1.5, color = gray(0.4)), part = "body") %>%

align(i = NULL, j = 1, align = "left", part = "all") %>%

align(i = NULL, j = 2:ncol(outdf), align = "center", part = "all") %>%

width(j = 1, width = 0.8) %>%

width(j = 2, width = 1.1) %>%

width(j = c(3:6, 9), width = 0.5) %>%

width(j = 7:8, width = 0.4) %>%

set_caption("Accuracy by Category")

Comparison with CT Predictions

{r

ctlocdf1 <- cttb2df0[, c("patno", "tuberculosis")]

ctlocdf2 <- locdf[, c("patno", "tuberculosis")]

colnames(ctlocdf1) <- c("patno", "tuberculosis1")

colnames(ctlocdf2) <- c("patno", "tuberculosis2")

ctlocdf <- merge(ctlocdf1, ctlocdf2, all.x = TRUE)

ctlocdf <- ctlocdf[!is.na(ctlocdf$tuberculosis2), ]

cor1 <- cor.test(ctlocdf$tuberculosis1, ctlocdf$tuberculosis2, method = "spearman")

For cases that include both CT images and CT localizer images, the predicted probability values for tuberculosis from each can be calculated. The correlation coefficient between the two was computed using Spearman's test, resulting in a correlation coefficient of r round(cor1$estimate[[1]], 3) and a P-value of r round(cor1$p.value[[1]], 3), indicating a significant correlation. The scatter plot is shown in Figure@ref(fig:scatter1).

{r

plot(tuberculosis2~tuberculosis1, data = ctlocdf, xlab = "CT", ylab = "CT Localizer")

Thus, CT localizer images can, to some extent, describe the imaging features of tuberculosis identified in CT scans.

Comparison with DR Predictions

{r

drloc_dr <- drdf[drdf$patno %in% locdf$patno & drdf$label %in% "tb", ]

drloc_normal <- drdf[drdf$label %in% "normal", ]

drloc_loc <- locdf[locdf$patno %in% drdf$patno, ]

drloc_dr <- drloc_dr[order(drloc_dr$patno), ]

drloc_loc <- drloc_loc[order(drloc_loc$patno), ]

# identical(drloc_dr$patno, drloc_loc$patno)

drloc_all <- data.frame(patno = c(drloc_dr$patno, drloc_normal$patno), tbdr = c(drloc_dr$tuberculosis, drloc_normal$tuberculosis), tbloc = c(drloc_loc$tuberculosis, drloc_normal$tuberculosis), label = c(drloc_dr$label, drloc_normal$label), airesdr = c(drloc_dr$aires, drloc_normal$aires), airesloc = c(drloc_loc$aires, drloc_normal$aires), case = c(drloc_dr$case, drloc_normal$case), lab = c(drloc_dr$lab, drloc_normal$lab), stringsAsFactors = FALSE)

cor2 <- cor.test(drloc_dr$tuberculosis, drloc_loc$tuberculosis, method = "spearman")

drloc_all$Y <- 1

drloc_all$Y[drloc_all$label %in% "normal"] <- 0

roc1 <- roc(drloc_all$Y, drloc_all$tbdr)

roc2 <- roc(drloc_all$Y, drloc_all$tbloc)

Since CT localizer images are derived from CT scans, and lung infection CT images were used as the control group in this study, the performance may be underestimated compared to using healthy individuals. We extracted cases that underwent both DR and CT examinations and had CT localizer images, totaling r nrow(drloc_loc) cases, all of which were tuberculosis cases.

The correlation coefficient between the two examination types was computed using Spearman's test, resulting in a correlation coefficient of r round(cor2$estimate[[1]], 3) and a P-value of r round(cor2$p.value[[1]], 3), indicating a significant correlation. The scatter plot is shown in Figure@ref(fig:scatter2).

{r

plot(x = drloc_dr$tuberculosis, y = drloc_loc$tuberculosis, xlab = "DR", ylab = "CT Localizer")

Based on the default thresholds for DR and CT localizer analysis, the prediction proportions for positive cases are shown in Table@ref(tab:disttbl4).

{r

outdf0 <- summarise(group_by(drloc_all[drloc_all$label %in% "tb", ], case, lab), num = length(patno), tb1 = sum(airesdr %in% "Active Pulmonary Tuberculosis"), abnorm1 = sum(!airesdr %in% "No Abnormalities"), tbrate1 = 0, abn1 = 0, tb2 = sum(airesloc %in% "Active Pulmonary Tuberculosis"), abnorm2 = sum(!airesloc %in% "No Abnormalities"), tbrate2 = 0, abn2 = 0)

outdf <- rbind(as.data.frame(lapply(outdf0[outdf0$case %in% "Confirmed Cases", ], FUN = function(X) ifelse(!inherits(X, "character"), sum(X), ifelse(grepl("Case", X[1]), X[1], "")))),

outdf0[outdf0$case %in% "Confirmed Cases", ],

as.data.frame(lapply(outdf0[outdf0$case %in% "Clinically Diagnosed Cases", ], FUN = function(X) ifelse(!inherits(X, "character"), sum(X), ifelse(grepl("Case", X[1]), X[1], "")))),

outdf0[outdf0$case %in% "Clinically Diagnosed Cases", ],

as.data.frame(lapply(outdf0[outdf0$case %in% "Suspected Cases", ], FUN = function(X) ifelse(!inherits(X, "character"), sum(X), ifelse(grepl("Case", X[1]), X[1], "")))),

outdf0[outdf0$case %in% "Suspected Cases", ],

as.data.frame(lapply(outdf0, FUN = function(X) ifelse(!inherits(X, "character"), sum(X), ""))))

outdf$case[nzchar(outdf$lab)] <- ""

outdf$case[nrow(outdf)] <- "Total"

outdf$tbrate1 <- paste0(format(outdf$tb1 / outdf$num * 100, digits = 1, nsmall = 1), "%")

outdf$abn1 <- paste0(format(outdf$abnorm1 / outdf$num * 100, digits = 1, nsmall = 1), "%")

outdf$tbrate2 <- paste0(format(outdf$tb2 / outdf$num * 100, digits = 1, nsmall = 1), "%")

outdf$abn2 <- paste0(format(outdf$abnorm2 / outdf$num * 100, digits = 1, nsmall = 1), "%")

colnames(outdf) <- c("Case Category", "Test Result", "Number of Cases", "DR Tuberculosis", "DR Abnormalities", "DR Tuberculosis Rate", "DR Abnormality Rate", "Localizer Tuberculosis", "Localizer Abnormalities", "Localizer Tuberculosis Rate", "Localizer Abnormality Rate")

regulartable(outdf) %>%

theme_vanilla() %>%

fontsize(size = 8, part = "header") %>%

fontsize(size = 7, part = "body") %>%

hline(i = c(sum(outdf0$case %in% "Confirmed Cases") + 1, sum(outdf0$case %in% c("Confirmed Cases", "Clinically Diagnosed Cases")) + 2, nrow(outdf)-1), j = NULL, border = fp_border(width=1.5, color = gray(0.4)), part = "body") %>%

align(i = NULL, j = 1, align = "left", part = "all") %>%

align(i = NULL, j = 2:ncol(outdf), align = "center", part = "all") %>%

width(j = 1, width = 0.8) %>%

width(j = 2, width = 1.1) %>%

width(j = c(6:7, 10:11), width = 0.5) %>%

width(j = c(4:5, 8:9), width = 0.4) %>%

set_caption("Accuracy by Category")

Currently, there are r nrow(drloc_loc) common tuberculosis cases for both DR and CT localizer images. Since the healthy individuals used as controls in DR analysis do not have CT localizer images, the predicted values for these negative samples on CT localizer images cannot be estimated. Assuming these negative samples had CT localizer images and their AI predictions were completely consistent with DR predictions, the ROC curves for DR and CT localizer images can be compared, as shown in Figure@ref(fig:roc5).

Based on the Youden index, the optimal threshold for DR was r round(coords(roc1, "best")[["threshold"]], 3), with a sensitivity of r round(coords(roc1, "best")[["sensitivity"]], 3) and a specificity of r round(coords(roc1, "best")[["specificity"]], 3). The optimal threshold for CT localizer images was r round(coords(roc2, "best")[["threshold"]][1], 3), with a sensitivity of r round(coords(roc2, "best")[["sensitivity"]][1], 3) and a specificity of r round(coords(roc2, "best")[["specificity"]][1], 3).

{r

plot(roc1, print.auc=TRUE, auc.polygon=TRUE,

grid.col=c("green", "red"), max.auc.polygon=TRUE,

auc.polygon.col="lightblue", print.thres=TRUE, xlim = c(1, 0))

plot(roc2, add = TRUE, col = "blue")

legend("bottomright", legend=c("CXR", "CT Localizer"),

col=c(par("fg"), "blue"), lwd=2)

Based on different thresholds, various accuracy metrics and their 95% confidence intervals for DR predictions are shown in Table@ref(tab:conftbl5).

{r

drloc_all$tb1 <- 0

drloc_all$tb1[drloc_all$tbdr > 0.35] <- 1

drloc_all$tb2 <- 0

drloc_all$tb2[drloc_all$tbdr > coords(roc1, "best")[["threshold"]]] <- 1

rocrpt0 <- reportROC(gold = as.factor(drloc_all$Y), predictor = drloc_all$tbdr, important = "se", plot = FALSE)

rocrpt1 <- reportROC(gold = as.factor(drloc_all$Y), predictor.binary = as.factor(drloc_all$tb1), important = "se", plot = FALSE)

rocrpt2 <- reportROC(gold = as.factor(drloc_all$Y), predictor.binary = as.factor(drloc_all$tb2), important = "se", plot = FALSE)

outdf <- data.frame(matrix(0, 2, 6))

outdf[[1]] <- c(0.35, round(coords(roc1, "best")[["threshold"]], 3))

outdf[1, 2] <- paste0(rocrpt0[["ACC"]], "\n(", rocrpt0[["ACC.low"]], " - ", rocrpt0[["ACC.up"]], ")")

outdf[1, 3] <- paste0(rocrpt1[["SEN"]], "\n(", rocrpt1[["SEN.low"]], " - ", rocrpt1[["SEN.up"]], ")")

outdf[1, 4] <- paste0(rocrpt1[["SPE"]], "\n(", rocrpt1[["SPE.low"]], " - ", rocrpt1[["SPE.up"]], ")")

outdf[1, 5] <- paste0(rocrpt0[["AUC"]], "\n(", rocrpt0[["AUC.low"]], " - ", rocrpt0[["AUC.up"]], ")")

outdf[1, 6] <- paste0(rocrpt0[["KAPPA"]], "\n(", rocrpt0[["KAPPA.low"]], " - ", rocrpt0[["KAPPA.up"]], ")")

outdf[2, 2] <- paste0(rocrpt0[["ACC"]], "\n(", rocrpt0[["ACC.low"]], " - ", rocrpt0[["ACC.up"]], ")")

outdf[2, 3] <- paste0(rocrpt2[["SEN"]], "\n(", rocrpt2[["SEN.low"]], " - ", rocrpt2[["SEN.up"]], ")")

outdf[2, 4] <- paste0(rocrpt2[["SPE"]], "\n(", rocrpt2[["SPE.low"]], " - ", rocrpt2[["SPE.up"]], ")")

outdf[2, 5] <- paste0(rocrpt0[["AUC"]], "\n(", rocrpt0[["AUC.low"]], " - ", rocrpt0[["AUC.up"]], ")")

outdf[2, 6] <- paste0(rocrpt0[["KAPPA"]], "\n(", rocrpt0[["KAPPA.low"]], " - ", rocrpt0[["KAPPA.up"]], ")")

colnames(outdf) <- c("Threshold", "Accuracy", "Sensitivity", "Specificity", "AUC", "Kappa Value")

regulartable(outdf) %>%

theme_vanilla() %>%

fontsize(size = 8, part = "header") %>%

fontsize(size = 7, part = "body") %>%

vline(i = NULL, j = c(1), border = fp_border(color="black"), part = "all") %>%

align(i = NULL, j = 1, align = "left", part = "all") %>%

align(i = NULL, j = 2:ncol(outdf), align = "center", part = "all") %>%

width(j = 1, width = 0.7) %>%

width(j = 2:ncol(outdf), width = 0.9) %>%

set_caption("Accuracy Metrics of DR Evaluation Results")

Based on different thresholds, various accuracy metrics and their 95% confidence intervals for CT localizer predictions are shown in Table@ref(tab:conftbl6).

{r

drloc_all$tb1 <- 0

drloc_all$tb1[drloc_all$tbloc > 0.35] <- 1

drloc_all$tb2 <- 0

drloc_all$tb2[drloc_all$tbloc > round(coords(roc2, "best")[["threshold"]][1], 3)] <- 1

rocrpt0 <- reportROC(gold = as.factor(drloc_all$Y), predictor = drloc_all$tbloc, important = "se", plot = FALSE)

rocrpt1 <- reportROC(gold = as.factor(drloc_all$Y), predictor.binary = as.factor(drloc_all$tb1), important = "se", plot = FALSE)

rocrpt2 <- reportROC(gold = as.factor(drloc_all$Y), predictor.binary = as.factor(drloc_all$tb2), important = "se", plot = FALSE)

outdf <- data.frame(matrix(0, 2, 6))

outdf[[1]] <- c(0.35, round(coords(roc2, "best")[["threshold"]][1], 3))

outdf[1, 2] <- paste0(rocrpt0[["ACC"]], "\n(", rocrpt0[["ACC.low"]], " - ", rocrpt0[["ACC.up"]], ")")

outdf[1, 3] <- paste0(rocrpt1[["SEN"]], "\n(", rocrpt1[["SEN.low"]], " - ", rocrpt1[["SEN.up"]], ")")

outdf[1, 4] <- paste0(rocrpt1[["SPE"]], "\n(", rocrpt1[["SPE.low"]], " - ", rocrpt1[["SPE.up"]], ")")

outdf[1, 5] <- paste0(rocrpt0[["AUC"]], "\n(", rocrpt0[["AUC.low"]], " - ", rocrpt0[["AUC.up"]], ")")

outdf[1, 6] <- paste0(rocrpt0[["KAPPA"]], "\n(", rocrpt0[["KAPPA.low"]], " - ", rocrpt0[["KAPPA.up"]], ")")

outdf[2, 2] <- paste0(rocrpt0[["ACC"]], "\n(", rocrpt0[["ACC.low"]], " - ", rocrpt0[["ACC.up"]], ")")

outdf[2, 3] <- paste0(rocrpt2[["SEN"]], "\n(", rocrpt2[["SEN.low"]], " - ", rocrpt2[["SEN.up"]], ")")

outdf[2, 4] <- paste0(rocrpt2[["SPE"]], "\n(", rocrpt2[["SPE.low"]], " - ", rocrpt2[["SPE.up"]], ")")

outdf[2, 5] <- paste0(rocrpt0[["AUC"]], "\n(", rocrpt0[["AUC.low"]], " - ", rocrpt0[["AUC.up"]], ")")

outdf[2, 6] <- paste0(rocrpt0[["KAPPA"]], "\n(", rocrpt0[["KAPPA.low"]], " - ", rocrpt0[["KAPPA.up"]], ")")

colnames(outdf) <- c("Threshold", "Accuracy", "Sensitivity", "Specificity", "AUC", "Kappa Value")

regulartable(outdf) %>%

theme_vanilla() %>%

fontsize(size = 8, part = "header") %>%

fontsize(size = 7, part = "body") %>%

vline(i = NULL, j = c(1), border = fp_border(color="black"), part = "all") %>%

align(i = NULL, j = 1, align = "left", part = "all") %>%

align(i = NULL, j = 2:ncol(outdf), align = "center", part = "all") %>%

width(j = 1, width = 0.7) %>%

width(j = 2:ncol(outdf), width = 0.9) %>%

set_caption("Accuracy Metrics of CT Localizer Evaluation Results")

We used the DeLong test to compare the ROC differences between the DR model and the CT localizer model, as shown in Table@ref(tab:delong1).

{r

test1 <- roc.test(roc1, roc2, method = "delong", alternative = "two.sided", paired = FALSE)

test2 <- roc.test(roc1, roc2, method = "delong", alternative = "less", paired = FALSE)

outdf <- data.frame(matrix(0, 2, 4))

outdf[[1]] <- c("two.sided", "less")

outdf[1, 2] <- round(test1$statistic[["D"]], 3)

outdf[1, 3] <- round(test1$parameter[["df"]], 2)

outdf[1, 4] <- round(test1$p.value, 4)

outdf[2, 2] <- round(test2$statistic[["D"]], 3)

outdf[2, 3] <- round(test2$parameter[["df"]], 2)

outdf[2, 4] <- round(test2$p.value, 4)

colnames(outdf) <- c("h1", "D", "df", "P Value")

regulartable(outdf) %>%

theme_vanilla() %>%

fontsize(size = 8, part = "header") %>%

fontsize(size = 7, part = "body") %>%

align(i = NULL, j = 1, align = "left", part = "all") %>%

align(i = NULL, j = 2:ncol(outdf), align = "center", part = "all") %>%

width(j = 1, width = 0.55) %>%

width(j = 2:ncol(outdf), width = 0.63) %>%

set_caption("DeLong Test")

Thus, the AUC of the DR model is lower than that of the CT localizer model, but the difference is not significant. It should be noted that the AUC value for CT localizer images may be overestimated, as the predicted values for negative samples are derived from DR data predictions, and in reality, negative samples do not have CT localizer images. However, based on experience, it is reasonable to believe that the AI has strong predictive ability for negative samples, so predictions based on CT localizer images have potential and deserve further study.
