# Supplementary material for: Validating the effectiveness of an AI algorithm for pulmonary tuberculosis screening using chest X-ray: Retrospective study and test accuracy with localizer images of the chest CT
Source: PLoS One. 2026 Feb 27;21(2):e0338810. doi: 10.1371/journal.pone.0338810 (PMC12948104; doi:10.1371/journal.pone.0338810)
Supplement: S1 File — Preprocessing scripts and model evaluation code are included. (ZIP) [file pone.0338810.s001.zip › CXR&Loc_code and data CHN original & ENG translation_anonymized/code and data Chinese original/Word version of the Rmd code.docx]

---

title: "肺结核AI分析"

author: "九峰医疗"

---

```{r data, include=FALSE}

library(dplyr)

library(kableExtra)

library(showtext)

library(flextable)

library(officer)

library(caret)

library(ggplot2)

library(readxl)

library(tmcn)

library(lubridate)

library(DiagrammeR)

library(htmlwidgets)

library(webshot)

library(caret)

library(pROC)

library(ggvenn)

library(reportROC)

drdf0 <- readRDS("../data/drdf.rds")

locdf0 <- readRDS("../data/locdf.rds")

cttb1df0 <- readRDS("../data/cttb1df.rds")

cttb2df0 <- readRDS("../data/cttb2df.rds")

patdf0 <- readRDS("../data/patdf.rds")

drdf <- drdf0

drdf$aires <- NA

drdf$aires[drdf$tuberculosis > 0.085] <- "活动性肺结核"

drdf$aires[is.na(drdf$aires) & drdf$old_tuberculosis > 0.5] <- "陈旧性肺结核"

drdf$aires[is.na(drdf$aires) & apply(drdf[, c("cardiomegaly", "pleural_disease", "mass", "pneumothorax", "pneumonia")], 1, max) > 0.5] <- "其他异常"

drdf$aires[is.na(drdf$aires)] <- "未见异常"

drdf$agegrp <- as.character(cut(drdf$age, breaks = c(-Inf, 20, 30, 40, 50, 60, 70, 80, 90, Inf)))

drdf <- merge(drdf, patdf0[, c("patno", "case", "lab")], all.x = TRUE)

locdf <- locdf0

locdf$aires <- NA

locdf$aires[locdf$tuberculosis > 0.35] <- "活动性肺结核"

locdf$aires[is.na(locdf$aires) & locdf$old_tuberculosis > 0.35] <- "陈旧性肺结核"

locdf$aires[is.na(locdf$aires) & apply(locdf[, c("cardiomegaly", "pleural_disease", "mass", "pneumothorax", "pneumonia")], 1, max) > 0.5] <- "其他异常"

locdf$aires[is.na(locdf$aires)] <- "未见异常"

locdf$agegrp <- as.character(cut(locdf$age, breaks = c(-Inf, 20, 30, 40, 50, 60, 70, 80, 90, Inf)))

locdf <- merge(locdf, patdf0[, c("patno", "case", "lab")], all.x = TRUE)

ctdf <- cttb1df0

ctdf$aires <- NA

ctdf$aires[ctdf$tuberculosis > 0.35] <- "活动性肺结核"

#ctdf$aires[is.na(ctdf$aires) & ctdf$infection > 0.5] <- "肺部感染"

ctdf$aires[is.na(ctdf$aires)] <- "未见异常"

ctdf$agegrp <- as.character(cut(ctdf$age, breaks = c(-Inf, 20, 30, 40, 50, 60, 70, 80, 90, Inf)))

ctdf <- merge(ctdf, patdf0[, c("patno", "case", "lab")], all.x = TRUE)

```

\mainmatter

# 数据概述

## 数据清洗

&emsp;&emsp;本次研究基于肺结核患者的DR影像及CT影像进行分析，使用程序扫描影像文件夹，自动识别DICOM格式的文件，然后运行九峰医疗的肺结核人工智能算法，得到AI预测活动性肺结核的概率。

&emsp;&emsp;对于同一个病案号包含多张影像的病例，取AI肺结核预测值最大的记录。数据中存在极少数病案号混淆的情况，予以剔除。

&emsp;&emsp;对于CT影像，提取5mm层厚平扫肺窗的影像进行AI分析，此外也提取定位片使用DR算法进行分析。

&emsp;&emsp;经过数据清洗后，共得到有效的DR胸片记录 `r nrow(drdf0)` 例，CT影像 `r nrow(cttb1df0)`

例，CT定位片 `r nrow(locdf0)` 例，样本之间的关系如图\@ref(fig:venn1)所示。

```{r venn1, fig.showtext = TRUE, fig.cap='样本详情', out.width='100%', fig.width=10, fig.asp=0.6, fig.align='center', echo=FALSE, message=FALSE, warning=FALSE}

vennlist <- list("CXR" = drdf0$patno, "CT Localizer" = locdf0$patno, "CT" = cttb1df0$patno)

ggvenn(vennlist, auto_scale = FALSE)

```

## 数据的分布情况

&emsp;&emsp;在包含DR片的`r nrow(drdf)`例病例中，平均年龄为 `r round(mean(drdf$age), 2)` 岁，男性患者有

`r sum(drdf$sex %in% "男")`

例（`r round(sum(drdf$sex %in% "男")/nrow(drdf)*100, 2)`\%），女性患者有

`r sum(drdf$sex %in% "女")`

例（`r round(sum(drdf$sex %in% "女")/nrow(drdf)*100, 2)`\%）。在包含CT影像的`r nrow(ctdf)`例病例中，平均年龄为

`r round(mean(ctdf$age), 2)` 岁，男性患者有 `r sum(ctdf$sex %in% "男")`

例（`r round(sum(ctdf$sex %in% "男")/nrow(ctdf)*100, 2)`\%），女性患者有

`r sum(ctdf$sex %in% "女")`

例（`r round(sum(ctdf$sex %in% "女")/nrow(ctdf)*100, 2)`\%）。在包含CT定位片的`r nrow(locdf)`例病例中，平均年龄为

`r round(mean(locdf$age), 2)` 岁，男性患者有 `r sum(locdf$sex %in% "男")`

例（`r round(sum(locdf$sex %in% "男")/nrow(locdf)*100, 2)`\%），女性患者有

`r sum(locdf$sex %in% "女")`

例（`r round(sum(locdf$sex %in% "女")/nrow(locdf)*100, 2)`\%）。病例的年龄分布如图\@ref(fig:distimg1)，年龄的分位数如表\@ref(tab:agetbl1)所示。

```{r distimg1, fig.showtext = TRUE, fig.cap='年龄分布', out.width='100%', fig.width=9, fig.asp=0.3, fig.align='center', echo=FALSE, message=FALSE, warning=FALSE}

par(mar = c(4, 4, 1, 1), mfrow = c(1, 3))

hist(drdf$age, xlab = "年龄（岁）", ylab = "频数", main = "DR")

hist(ctdf$age, xlab = "年龄（岁）", ylab = "频数", main = "CT")

hist(locdf$age, xlab = "年龄（岁）", ylab = "频数", main = "CT定位片")

```

```{r agetbl1, echo=FALSE, message=FALSE,results='asis',warning=FALSE}

outdf <- data.frame(matrix(0, 4, 6))

outdf[[1]] <- c("DR", "CT", "CT定位片", "汇总")

outdf[1, 2:6] <- fivenum(drdf$age, na.rm = TRUE)

outdf[2, 2:6] <- fivenum(ctdf$age, na.rm = TRUE)

outdf[3, 2:6] <- fivenum(locdf$age, na.rm = TRUE)

outdf[4, 2:6] <- fivenum(c(drdf$age, ctdf$age, locdf$age), na.rm = TRUE)

colnames(outdf) <- c("样本类型", "最小值", "下四分位数", "中位数", "上四分位数", "最大值")

regulartable(outdf) %>%

theme_vanilla() %>%

fontsize(size = 8, part = "header") %>%

fontsize(size = 7, part = "body") %>%

vline(i = NULL, j = c(1), border = fp_border(color="black"), part = "all") %>%

hline(i = nrow(outdf)-1, j = NULL, border = fp_border(width=1.5, color=gray(0.4)), part = "body") %>%

align(i = NULL, j = 1, align = "left", part = "all") %>%

align(i = NULL, j = 2:ncol(outdf), align = "center", part = "all") %>%

width(j = 1, width = 0.6) %>%

width(j = 2:ncol(outdf), width = 0.7) %>%

set_caption("年龄的分位数")

```

# DR影像分析

## 基于DR模型的预测准确率分析

&emsp;&emsp;在本次研究中以健康人的体检DR胸片为对照组，共得到`r sum(drdf$label %in% "normal")`例样本，对照组的平均年龄为

`r round(mean(drdf$age[drdf$label %in% "normal"]), 2)` 岁，男性患者有 `r sum(drdf$label %in% "normal" & drdf$sex %in% "男")`

例（`r round(sum(drdf$label %in% "normal" & drdf$sex %in% "男")/sum(drdf$label %in% "normal")*100, 2)`\%），女性患者有

`r sum(drdf$label %in% "normal" & drdf$sex %in% "女")`

例（`r round(sum(drdf$label %in% "normal" & drdf$sex %in% "女")/sum(drdf$label %in% "normal")*100, 2)`\%）。阳性样本的平均年龄为

`r round(mean(drdf$age[drdf$label %in% "tb"]), 2)` 岁，男性患者有

`r sum(drdf$label %in% "tb" & drdf$sex %in% "男")`

例（`r round(sum(drdf$label %in% "tb" & drdf$sex %in% "男")/sum(drdf$label %in% "tb")*100, 2)`\%），女性患者有

`r sum(drdf$label %in% "tb" & drdf$sex %in% "女")`

例（`r round(sum(drdf$label %in% "tb" & drdf$sex %in% "女")/sum(drdf$label %in% "tb")*100, 2)`\%）。不同样本组年龄分布如图\@ref(fig:distsamp1)，年龄的分位数如表\@ref(tab:agesamp1)所示。

```{r distsamp1, fig.showtext = TRUE, fig.cap='年龄分布', out.width='100%', fig.width=12, fig.heigth=6, fig.align='center', echo=FALSE, message=FALSE, warning=FALSE}

par(mar = c(4, 4, 1, 1), mfrow = c(1, 2))

hist(drdf$age[drdf$label %in% "normal"], xlab = "年龄（岁）", ylab = "频数", main = "阴性样本")

hist(drdf$age[drdf$label %in% "tb"], xlab = "年龄（岁）", ylab = "频数", main = "阳性样本")

```

```{r agesamp1, echo=FALSE, message=FALSE,results='asis',warning=FALSE}

outdf <- data.frame(matrix(0, 3, 6))

outdf[[1]] <- c("阴性", "阳性", "汇总")

outdf[2, 2:6] <- fivenum(drdf$age[drdf$label %in% "normal"], na.rm = TRUE)

outdf[1, 2:6] <- fivenum(drdf$age[drdf$label %in% "tb"], na.rm = TRUE)

outdf[3, 2:6] <- fivenum(drdf$age, na.rm = TRUE)

colnames(outdf) <- c("样本类型", "最小值", "下四分位数", "中位数", "上四分位数", "最大值")

regulartable(outdf) %>%

theme_vanilla() %>%

fontsize(size = 8, part = "header") %>%

fontsize(size = 7, part = "body") %>%

vline(i = NULL, j = c(1), border = fp_border(color="black"), part = "all") %>%

hline(i = nrow(outdf)-1, j = NULL, border = fp_border(width=1.5, color=gray(0.4)), part = "body") %>%

align(i = NULL, j = 1, align = "left", part = "all") %>%

align(i = NULL, j = 2:ncol(outdf), align = "center", part = "all") %>%

width(j = 1, width = 0.6) %>%

width(j = 2:ncol(outdf), width = 0.7) %>%

set_caption("年龄的分位数")

drdf$Y <- 1

drdf$Y[drdf$label %in% "normal"] <- 0

roc1 <- roc(drdf$Y, drdf$tuberculosis)

```

&emsp;&emsp;以本研究及测试集中诊断结果为金标准，使用基于DR的AI算法进行预测，绘制AI预测结果的ROC曲线，如图\@ref(fig:roc1)所示，

在定诊病例数据集上，AI的最佳阈值为 `r round(coords(roc1, "best")[["threshold"]], 3)`，计算得到的灵敏度为

`r round(coords(roc1, "best")[["sensitivity"]], 3)`，特异度为

`r round(coords(roc1, "best")[["specificity"]], 3)`。

```{r roc1, fig.showtext = TRUE, fig.cap= 'AI评估结果的ROC曲线', out.width='90%', fig.width=8, fig.asp=0.9, fig.align='center', echo=FALSE, message=FALSE}

plot(roc1, print.auc=TRUE, auc.polygon=TRUE, grid=c(0.1, 0.2),

grid.col=c("green", "red"), max.auc.polygon=TRUE,

auc.polygon.col="lightblue", print.thres=TRUE)

```

&emsp;&emsp;基于不同的阈值，计算各准确性指标及其95\%置信区间，结果如表\@ref(tab:conftbl1)所示。

```{r conftbl1, echo=FALSE, message=FALSE,results='asis',warning=FALSE}

drdf$tb1 <- 0

drdf$tb1[drdf$tuberculosis > 0.35] <- 1

drdf$tb2 <- 0

drdf$tb2[drdf$tuberculosis > coords(roc1, "best")[["threshold"]]] <- 1

rocrpt0 <- reportROC(gold = as.factor(drdf$Y), predictor = drdf$tuberculosis, important = "se", plot = FALSE)

rocrpt1 <- reportROC(gold = as.factor(drdf$Y), predictor.binary = as.factor(drdf$tb1), important = "se", plot = FALSE)

rocrpt2 <- reportROC(gold = as.factor(drdf$Y), predictor.binary = as.factor(drdf$tb2), important = "se", plot = FALSE)

outdf <- data.frame(matrix(0, 2, 6))

outdf[[1]] <- c(0.35, round(coords(roc1, "best")[["threshold"]], 3))

outdf[1, 2] <- paste0(rocrpt0[["ACC"]], "\n(", rocrpt0[["ACC.low"]], " - ", rocrpt0[["ACC.up"]], ")")

outdf[1, 3] <- paste0(rocrpt1[["SEN"]], "\n(", rocrpt1[["SEN.low"]], " - ", rocrpt1[["SEN.up"]], ")")

outdf[1, 4] <- paste0(rocrpt1[["SPE"]], "\n(", rocrpt1[["SPE.low"]], " - ", rocrpt1[["SPE.up"]], ")")

outdf[1, 5] <- paste0(rocrpt0[["AUC"]], "\n(", rocrpt0[["AUC.low"]], " - ", rocrpt0[["AUC.up"]], ")")

outdf[1, 6] <- paste0(rocrpt0[["KAPPA"]], "\n(", rocrpt0[["KAPPA.low"]], " - ", rocrpt0[["KAPPA.up"]], ")")

outdf[2, 2] <- paste0(rocrpt0[["ACC"]], "\n(", rocrpt0[["ACC.low"]], " - ", rocrpt0[["ACC.up"]], ")")

outdf[2, 3] <- paste0(rocrpt2[["SEN"]], "\n(", rocrpt2[["SEN.low"]], " - ", rocrpt2[["SEN.up"]], ")")

outdf[2, 4] <- paste0(rocrpt2[["SPE"]], "\n(", rocrpt2[["SPE.low"]], " - ", rocrpt2[["SPE.up"]], ")")

outdf[2, 5] <- paste0(rocrpt0[["AUC"]], "\n(", rocrpt0[["AUC.low"]], " - ", rocrpt0[["AUC.up"]], ")")

outdf[2, 6] <- paste0(rocrpt0[["KAPPA"]], "\n(", rocrpt0[["KAPPA.low"]], " - ", rocrpt0[["KAPPA.up"]], ")")

colnames(outdf) <- c("阈值", "准确率", "灵敏度", "特异度", "AUC", "Kappa值")

regulartable(outdf) %>%

theme_vanilla() %>%

fontsize(size = 8, part = "header") %>%

fontsize(size = 7, part = "body") %>%

vline(i = NULL, j = c(1), border = fp_border(color="black"), part = "all") %>%

align(i = NULL, j = 1, align = "left", part = "all") %>%

align(i = NULL, j = 2:ncol(outdf), align = "center", part = "all") %>%

width(j = 1, width = 0.7) %>%

width(j = 2:ncol(outdf), width = 0.9) %>%

set_caption("AI评估结果的准确性指标")

```

## 对阳性病例的预测结果分析

&emsp;&emsp;本次研究主要关注活动性肺结核的预测准确率，但AI除了输出活动性肺结核的预测概率以外，还会输出陈旧性肺结核、肺炎、气胸、结节肿块、胸膜病变、心影增大等异常，我们将预测结果分为四类：活动性肺结核、陈旧性肺结核、其他异常、未见异常。针对不同的诊断结果，分别统计其预测比例，如表

\@ref(tab:disttbl1)所示。

```{r disttbl1, echo=FALSE, message=FALSE,results='asis',warning=FALSE}

outdf0 <- summarise(group_by(drdf[drdf$label %in% "tb", ], case, lab), num = length(patno), tb = sum(aires %in% "活动性肺结核"), tbrate = 0, oldtb = sum(aires %in% "陈旧性肺结核"), other = sum(aires %in% "其他异常"), norm = sum(aires %in% "未见异常"))

outdf <- rbind(as.data.frame(lapply(outdf0[outdf0$case %in% "确诊病例", ], FUN = function(X) ifelse(!inherits(X, "character"), sum(X), ifelse(grepl("病例", X[1]), X[1], "")))),

outdf0[outdf0$case %in% "确诊病例", ],

as.data.frame(lapply(outdf0[outdf0$case %in% "临床诊断病例", ], FUN = function(X) ifelse(!inherits(X, "character"), sum(X), ifelse(grepl("病例", X[1]), X[1], "")))),

outdf0[outdf0$case %in% "临床诊断病例", ],

as.data.frame(lapply(outdf0[outdf0$case %in% "疑似病例", ], FUN = function(X) ifelse(!inherits(X, "character"), sum(X), ifelse(grepl("病例", X[1]), X[1], "")))),

outdf0[outdf0$case %in% "疑似病例", ],

as.data.frame(lapply(outdf0, FUN = function(X) ifelse(!inherits(X, "character"), sum(X), ""))))

outdf$case[nzchar(outdf$lab)] <- ""

outdf$case[nrow(outdf)] <- "汇总"

outdf$tbrate <- paste0(format(outdf$tb / outdf$num * 100, digits = 1, nsmall = 1), "%")

outdf$abn <- paste0(format(100 - outdf$norm / outdf$num * 100, digits = 1, nsmall = 1), "%")

colnames(outdf) <- c("病例分类", "检测结果", "病例数", "活动性肺结核", "肺结核阳性率", "陈旧性肺结核", "其他异常", "未见异常", "异常率")

regulartable(outdf) %>%

theme_vanilla() %>%

fontsize(size = 8, part = "header") %>%

fontsize(size = 7, part = "body") %>%

hline(i = c(sum(outdf0$case %in% "确诊病例") + 1, sum(outdf0$case %in% c("确诊病例", "临床诊断病例")) + 2, nrow(outdf)-1), j = NULL, border = fp_border(width=1.5, color = gray(0.4)), part = "body") %>%

align(i = NULL, j = 1, align = "left", part = "all") %>%

align(i = NULL, j = 2:ncol(outdf), align = "center", part = "all") %>%

width(j = 1, width = 0.8) %>%

width(j = 2, width = 1.1) %>%

width(j = c(3:6, 9), width = 0.5) %>%

width(j = 7:8, width = 0.4) %>%

set_caption("各类别准确率")

```

# CT影像分析

## 基于CT模型的预测准确率分析

&emsp;&emsp;在本次研究中以肺部感染的CT影像为对照组，共得到`r sum(ctdf$label %in% "infection")`例样本，对照组的平均年龄为

`r round(mean(ctdf$age[ctdf$label %in% "infection"]), 2)` 岁，男性患者有

`r sum(ctdf$label %in% "infection" & ctdf$sex %in% "男")`

例（`r round(sum(ctdf$label %in% "infection" & ctdf$sex %in% "男")/sum(ctdf$label %in% "infection")*100, 2)`\%），女性患者有

`r sum(ctdf$label %in% "infection" & ctdf$sex %in% "女")`

例（`r round(sum(ctdf$label %in% "infection" & ctdf$sex %in% "女")/sum(ctdf$label %in% "infection")*100, 2)`\%）。阳性样本的平均年龄为

`r round(mean(ctdf$age[ctdf$label %in% "tb"]), 2)` 岁，男性患者有

`r sum(ctdf$label %in% "tb" & ctdf$sex %in% "男")`

例（`r round(sum(ctdf$label %in% "tb" & ctdf$sex %in% "男")/sum(ctdf$label %in% "tb")*100, 2)`\%），女性患者有

`r sum(ctdf$label %in% "tb" & ctdf$sex %in% "女")`

例（`r round(sum(ctdf$label %in% "tb" & ctdf$sex %in% "女")/sum(ctdf$label %in% "tb")*100, 2)`\%）。不同样本组年龄分布如图\@ref(fig:distsamp2)，年龄的分位数如表\@ref(tab:agesamp2)所示。

```{r distsamp2, fig.showtext = TRUE, fig.cap='年龄分布', out.width='100%', fig.width=12, fig.heigth=6, fig.align='center', echo=FALSE, message=FALSE, warning=FALSE}

par(mar = c(4, 4, 1, 1), mfrow = c(1, 2))

hist(ctdf$age[ctdf$label %in% "infection"], xlab = "年龄（岁）", ylab = "频数", main = "对照样本")

hist(ctdf$age[ctdf$label %in% "tb"], xlab = "年龄（岁）", ylab = "频数", main = "阳性样本")

```

```{r agesamp2, echo=FALSE, message=FALSE,results='asis',warning=FALSE}

outdf <- data.frame(matrix(0, 3, 6))

outdf[[1]] <- c("对照", "阳性", "汇总")

outdf[2, 2:6] <- fivenum(ctdf$age[ctdf$label %in% "infection"], na.rm = TRUE)

outdf[1, 2:6] <- fivenum(ctdf$age[ctdf$label %in% "tb"], na.rm = TRUE)

outdf[3, 2:6] <- fivenum(ctdf$age, na.rm = TRUE)

colnames(outdf) <- c("样本类型", "最小值", "下四分位数", "中位数", "上四分位数", "最大值")

regulartable(outdf) %>%

theme_vanilla() %>%

fontsize(size = 8, part = "header") %>%

fontsize(size = 7, part = "body") %>%

vline(i = NULL, j = c(1), border = fp_border(color="black"), part = "all") %>%

hline(i = nrow(outdf)-1, j = NULL, border = fp_border(width=1.5, color=gray(0.4)), part = "body") %>%

align(i = NULL, j = 1, align = "left", part = "all") %>%

align(i = NULL, j = 2:ncol(outdf), align = "center", part = "all") %>%

width(j = 1, width = 0.6) %>%

width(j = 2:ncol(outdf), width = 0.7) %>%

set_caption("年龄的分位数")

ctdf$Y <- 1

ctdf$Y[ctdf$label %in% "infection"] <- 0

roc1 <- roc(ctdf$Y, ctdf$tuberculosis)

```

&emsp;&emsp;以本研究及测试集中诊断结果为金标准，使用基于CT的AI算法进行预测，绘制AI预测结果的ROC曲线，如图\@ref(fig:roc2)所示，

在定诊病例数据集上，AI的最佳阈值为 `r round(coords(roc1, "best")[["threshold"]], 3)`，计算得到的灵敏度为

`r round(coords(roc1, "best")[["sensitivity"]], 3)`，特异度为

`r round(coords(roc1, "best")[["specificity"]], 3)`。

```{r roc2, fig.showtext = TRUE, fig.cap= 'AI评估结果的ROC曲线', out.width='90%', fig.width=8, fig.asp=0.9, fig.align='center', echo=FALSE, message=FALSE}

plot(roc1, print.auc=TRUE, auc.polygon=TRUE, grid=c(0.1, 0.2),

grid.col=c("green", "red"), max.auc.polygon=TRUE,

auc.polygon.col="lightblue", print.thres=TRUE)

```

&emsp;&emsp;基于不同的阈值，计算各准确性指标及其95\%置信区间，结果如表\@ref(tab:conftbl2)所示。

```{r conftbl2, echo=FALSE, message=FALSE,results='asis',warning=FALSE}

ctdf$tb1 <- 0

ctdf$tb1[ctdf$tuberculosis > 0.5] <- 1

ctdf$tb2 <- 0

ctdf$tb2[ctdf$tuberculosis > coords(roc1, "best")[["threshold"]]] <- 1

rocrpt0 <- reportROC(gold = as.factor(ctdf$Y), predictor = ctdf$tuberculosis, important = "se", plot = FALSE)

rocrpt1 <- reportROC(gold = as.factor(ctdf$Y), predictor.binary = as.factor(ctdf$tb1), important = "se", plot = FALSE)

rocrpt2 <- reportROC(gold = as.factor(ctdf$Y), predictor.binary = as.factor(ctdf$tb2), important = "se", plot = FALSE)

outdf <- data.frame(matrix(0, 2, 6))

outdf[[1]] <- c(0.5, round(coords(roc1, "best")[["threshold"]], 3))

outdf[1, 2] <- paste0(rocrpt0[["ACC"]], "\n(", rocrpt0[["ACC.low"]], " - ", rocrpt0[["ACC.up"]], ")")

outdf[1, 3] <- paste0(rocrpt1[["SEN"]], "\n(", rocrpt1[["SEN.low"]], " - ", rocrpt1[["SEN.up"]], ")")

outdf[1, 4] <- paste0(rocrpt1[["SPE"]], "\n(", rocrpt1[["SPE.low"]], " - ", rocrpt1[["SPE.up"]], ")")

outdf[1, 5] <- paste0(rocrpt0[["AUC"]], "\n(", rocrpt0[["AUC.low"]], " - ", rocrpt0[["AUC.up"]], ")")

outdf[1, 6] <- paste0(rocrpt0[["KAPPA"]], "\n(", rocrpt0[["KAPPA.low"]], " - ", rocrpt0[["KAPPA.up"]], ")")

outdf[2, 2] <- paste0(rocrpt0[["ACC"]], "\n(", rocrpt0[["ACC.low"]], " - ", rocrpt0[["ACC.up"]], ")")

outdf[2, 3] <- paste0(rocrpt2[["SEN"]], "\n(", rocrpt2[["SEN.low"]], " - ", rocrpt2[["SEN.up"]], ")")

outdf[2, 4] <- paste0(rocrpt2[["SPE"]], "\n(", rocrpt2[["SPE.low"]], " - ", rocrpt2[["SPE.up"]], ")")

outdf[2, 5] <- paste0(rocrpt0[["AUC"]], "\n(", rocrpt0[["AUC.low"]], " - ", rocrpt0[["AUC.up"]], ")")

outdf[2, 6] <- paste0(rocrpt0[["KAPPA"]], "\n(", rocrpt0[["KAPPA.low"]], " - ", rocrpt0[["KAPPA.up"]], ")")

colnames(outdf) <- c("阈值", "准确率", "灵敏度", "特异度", "AUC", "Kappa值")

regulartable(outdf) %>%

theme_vanilla() %>%

fontsize(size = 8, part = "header") %>%

fontsize(size = 7, part = "body") %>%

vline(i = NULL, j = c(1), border = fp_border(color="black"), part = "all") %>%

align(i = NULL, j = 1, align = "left", part = "all") %>%

align(i = NULL, j = 2:ncol(outdf), align = "center", part = "all") %>%

width(j = 1, width = 0.7) %>%

width(j = 2:ncol(outdf), width = 0.9) %>%

set_caption("AI评估结果的准确性指标")

```

## 优化后的CT模型预测准确率分析

```{r sample3, include=FALSE}

ctdf <- cttb2df0

ctdf$aires <- NA

ctdf$aires[ctdf$tuberculosis > 0.938] <- "活动性肺结核"

ctdf$aires[is.na(ctdf$aires)] <- "其他"

ctdf$agegrp <- as.character(cut(ctdf$age, breaks = c(-Inf, 20, 30, 40, 50, 60, 70, 80, 90, Inf)))

ctdf <- merge(ctdf, patdf0[, c("patno", "case", "lab")], all.x = TRUE)

ctdf$Y <- 1

ctdf$Y[ctdf$label %in% "infection"] <- 0

roc1 <- roc(ctdf$Y, ctdf$tuberculosis)

```

&emsp;&emsp;使用优化后的AI算法进行预测，用5折交叉验证的结果进行评估，绘制AI预测结果的ROC曲线，如图\@ref(fig:roc3)所示，

在定诊病例数据集上，AI的最佳阈值为 `r round(coords(roc1, "best")[["threshold"]], 3)`，计算得到的灵敏度为

`r round(coords(roc1, "best")[["sensitivity"]], 3)`，特异度为

`r round(coords(roc1, "best")[["specificity"]], 3)`。

```{r roc3, fig.showtext = TRUE, fig.cap= 'AI评估结果的ROC曲线', out.width='90%', fig.width=8, fig.asp=0.9, fig.align='center', echo=FALSE, message=FALSE}

plot(roc1, print.auc=TRUE, auc.polygon=TRUE, grid=c(0.1, 0.2),

grid.col=c("green", "red"), max.auc.polygon=TRUE,

auc.polygon.col="lightblue", print.thres=TRUE)

```

&emsp;&emsp;基于不同的阈值，计算各准确性指标及其95\%置信区间，结果如表\@ref(tab:conftbl3)所示。

```{r conftbl3, echo=FALSE, message=FALSE,results='asis',warning=FALSE}

ctdf$tb1 <- 0

ctdf$tb1[ctdf$tuberculosis > 0.5] <- 1

ctdf$tb2 <- 0

ctdf$tb2[ctdf$tuberculosis > coords(roc1, "best")[["threshold"]]] <- 1

rocrpt0 <- reportROC(gold = as.factor(ctdf$Y), predictor = ctdf$tuberculosis, important = "se", plot = FALSE)

rocrpt1 <- reportROC(gold = as.factor(ctdf$Y), predictor.binary = as.factor(ctdf$tb1), important = "se", plot = FALSE)

rocrpt2 <- reportROC(gold = as.factor(ctdf$Y), predictor.binary = as.factor(ctdf$tb2), important = "se", plot = FALSE)

outdf <- data.frame(matrix(0, 2, 6))

outdf[[1]] <- c(0.5, round(coords(roc1, "best")[["threshold"]], 3))

outdf[1, 2] <- paste0(rocrpt0[["ACC"]], "\n(", rocrpt0[["ACC.low"]], " - ", rocrpt0[["ACC.up"]], ")")

outdf[1, 3] <- paste0(rocrpt1[["SEN"]], "\n(", rocrpt1[["SEN.low"]], " - ", rocrpt1[["SEN.up"]], ")")

outdf[1, 4] <- paste0(rocrpt1[["SPE"]], "\n(", rocrpt1[["SPE.low"]], " - ", rocrpt1[["SPE.up"]], ")")

outdf[1, 5] <- paste0(rocrpt0[["AUC"]], "\n(", rocrpt0[["AUC.low"]], " - ", rocrpt0[["AUC.up"]], ")")

outdf[1, 6] <- paste0(rocrpt0[["KAPPA"]], "\n(", rocrpt0[["KAPPA.low"]], " - ", rocrpt0[["KAPPA.up"]], ")")

outdf[2, 2] <- paste0(rocrpt0[["ACC"]], "\n(", rocrpt0[["ACC.low"]], " - ", rocrpt0[["ACC.up"]], ")")

outdf[2, 3] <- paste0(rocrpt2[["SEN"]], "\n(", rocrpt2[["SEN.low"]], " - ", rocrpt2[["SEN.up"]], ")")

outdf[2, 4] <- paste0(rocrpt2[["SPE"]], "\n(", rocrpt2[["SPE.low"]], " - ", rocrpt2[["SPE.up"]], ")")

outdf[2, 5] <- paste0(rocrpt0[["AUC"]], "\n(", rocrpt0[["AUC.low"]], " - ", rocrpt0[["AUC.up"]], ")")

outdf[2, 6] <- paste0(rocrpt0[["KAPPA"]], "\n(", rocrpt0[["KAPPA.low"]], " - ", rocrpt0[["KAPPA.up"]], ")")

colnames(outdf) <- c("阈值", "准确率", "灵敏度", "特异度", "AUC", "Kappa值")

regulartable(outdf) %>%

theme_vanilla() %>%

fontsize(size = 8, part = "header") %>%

fontsize(size = 7, part = "body") %>%

vline(i = NULL, j = c(1), border = fp_border(color="black"), part = "all") %>%

align(i = NULL, j = 1, align = "left", part = "all") %>%

align(i = NULL, j = 2:ncol(outdf), align = "center", part = "all") %>%

width(j = 1, width = 0.7) %>%

width(j = 2:ncol(outdf), width = 0.9) %>%

set_caption("AI评估结果的准确性指标")

```

## 对阳性病例的预测结果分析

&emsp;&emsp;本次研究主要关注活动性肺结核的预测准确率，和肺部感染进行鉴别诊断，针对不同类别分别计算预测准确率，如表

\@ref(tab:disttbl2)所示。

```{r disttbl2, echo=FALSE, message=FALSE,results='asis',warning=FALSE}

outdf0 <- summarise(group_by(ctdf[ctdf$label %in% "tb", ], case, lab), num = length(patno), tb = sum(aires %in% "活动性肺结核"), tbrate = 0)

outdf <- rbind(as.data.frame(lapply(outdf0[outdf0$case %in% "确诊病例", ], FUN = function(X) ifelse(!inherits(X, "character"), sum(X), ifelse(grepl("病例", X[1]), X[1], "")))),

outdf0[outdf0$case %in% "确诊病例", ],

as.data.frame(lapply(outdf0[outdf0$case %in% "临床诊断病例", ], FUN = function(X) ifelse(!inherits(X, "character"), sum(X), ifelse(grepl("病例", X[1]), X[1], "")))),

outdf0[outdf0$case %in% "临床诊断病例", ],

as.data.frame(lapply(outdf0[outdf0$case %in% "疑似病例", ], FUN = function(X) ifelse(!inherits(X, "character"), sum(X), ifelse(grepl("病例", X[1]), X[1], "")))),

outdf0[outdf0$case %in% "疑似病例", ],

as.data.frame(lapply(outdf0, FUN = function(X) ifelse(!inherits(X, "character"), sum(X), ""))))

outdf$case[nzchar(outdf$lab)] <- ""

outdf$case[nrow(outdf)] <- "汇总"

outdf$tbrate <- paste0(format(outdf$tb / outdf$num * 100, digits = 1, nsmall = 1), "%")

colnames(outdf) <- c("病例分类", "检测结果", "病例数", "活动性肺结核", "肺结核阳性率")

regulartable(outdf) %>%

theme_vanilla() %>%

fontsize(size = 8, part = "header") %>%

fontsize(size = 7, part = "body") %>%

hline(i = c(sum(outdf0$case %in% "确诊病例") + 1, sum(outdf0$case %in% c("确诊病例", "临床诊断病例")) + 2, nrow(outdf)-1), j = NULL, border = fp_border(width=1.5, color = gray(0.4)), part = "body") %>%

align(i = NULL, j = 1, align = "left", part = "all") %>%

align(i = NULL, j = 2:ncol(outdf), align = "center", part = "all") %>%

width(j = 1, width = 0.8) %>%

width(j = 2, width = 1.1) %>%

width(j = c(3:5), width = 0.5) %>%

set_caption("各类别准确率")

```

# CT定位片分析

## 基于DR模型的预测准确率分析

&emsp;&emsp;在本次研究中以肺部感染的CT定位片为对照组，共得到`r sum(locdf$label %in% "infection")`例样本，对照组的平均年龄为

`r round(mean(locdf$age[locdf$label %in% "infection"]), 2)` 岁，男性患者有

`r sum(locdf$label %in% "infection" & locdf$sex %in% "男")`

例（`r round(sum(locdf$label %in% "infection" & locdf$sex %in% "男")/sum(locdf$label %in% "infection")*100, 2)`\%），女性患者有

`r sum(locdf$label %in% "infection" & locdf$sex %in% "女")`

例（`r round(sum(locdf$label %in% "infection" & locdf$sex %in% "女")/sum(locdf$label %in% "infection")*100, 2)`\%）。阳性样本的平均年龄为

`r round(mean(locdf$age[locdf$label %in% "tb"]), 2)` 岁，男性患者有

`r sum(locdf$label %in% "tb" & locdf$sex %in% "男")`

例（`r round(sum(locdf$label %in% "tb" & locdf$sex %in% "男")/sum(locdf$label %in% "tb")*100, 2)`\%），女性患者有

`r sum(locdf$label %in% "tb" & locdf$sex %in% "女")`

例（`r round(sum(locdf$label %in% "tb" & locdf$sex %in% "女")/sum(locdf$label %in% "tb")*100, 2)`\%）。不同样本组年龄分布如图\@ref(fig:distsamp3)，年龄的分位数如表\@ref(tab:agesamp3)所示。

```{r distsamp3, fig.showtext = TRUE, fig.cap='年龄分布', out.width='100%', fig.width=12, fig.heigth=6, fig.align='center', echo=FALSE, message=FALSE, warning=FALSE}

par(mar = c(4, 4, 1, 1), mfrow = c(1, 2))

hist(locdf$age[locdf$label %in% "infection"], xlab = "年龄（岁）", ylab = "频数", main = "阴性样本")

hist(locdf$age[locdf$label %in% "tb"], xlab = "年龄（岁）", ylab = "频数", main = "阳性样本")

```

```{r agesamp3, echo=FALSE, message=FALSE,results='asis',warning=FALSE}

outdf <- data.frame(matrix(0, 3, 6))

outdf[[1]] <- c("阴性", "阳性", "汇总")

outdf[2, 2:6] <- fivenum(locdf$age[locdf$label %in% "infection"], na.rm = TRUE)

outdf[1, 2:6] <- fivenum(locdf$age[locdf$label %in% "tb"], na.rm = TRUE)

outdf[3, 2:6] <- fivenum(locdf$age, na.rm = TRUE)

colnames(outdf) <- c("样本类型", "最小值", "下四分位数", "中位数", "上四分位数", "最大值")

regulartable(outdf) %>%

theme_vanilla() %>%

fontsize(size = 8, part = "header") %>%

fontsize(size = 7, part = "body") %>%

vline(i = NULL, j = c(1), border = fp_border(color="black"), part = "all") %>%

hline(i = nrow(outdf)-1, j = NULL, border = fp_border(width=1.5, color=gray(0.4)), part = "body") %>%

align(i = NULL, j = 1, align = "left", part = "all") %>%

align(i = NULL, j = 2:ncol(outdf), align = "center", part = "all") %>%

width(j = 1, width = 0.6) %>%

width(j = 2:ncol(outdf), width = 0.7) %>%

set_caption("年龄的分位数")

locdf$Y <- 1

locdf$Y[locdf$label %in% "infection"] <- 0

roc1 <- roc(locdf$Y, locdf$tuberculosis)

```

&emsp;&emsp;以本研究及测试集中诊断结果为金标准，使用基于DR的AI算法进行预测，绘制AI预测结果的ROC曲线，如图\@ref(fig:roc4)所示，

在定诊病例数据集上，AI的最佳阈值为 `r round(coords(roc1, "best")[["threshold"]], 3)`，计算得到的灵敏度为

`r round(coords(roc1, "best")[["sensitivity"]], 3)`，特异度为

`r round(coords(roc1, "best")[["specificity"]], 3)`。

```{r roc4, fig.showtext = TRUE, fig.cap= 'AI评估结果的ROC曲线', out.width='90%', fig.width=8, fig.asp=0.9, fig.align='center', echo=FALSE, message=FALSE}

plot(roc1, print.auc=TRUE, auc.polygon=TRUE, grid=c(0.1, 0.2),

grid.col=c("green", "red"), max.auc.polygon=TRUE,

auc.polygon.col="lightblue", print.thres=TRUE)

```

&emsp;&emsp;基于不同的阈值，计算各准确性指标及其95\%置信区间，结果如表\@ref(tab:conftbl4)所示。

```{r conftbl4, echo=FALSE, message=FALSE,results='asis',warning=FALSE}

locdf$tb1 <- 0

locdf$tb1[locdf$tuberculosis > 0.35] <- 1

locdf$tb2 <- 0

locdf$tb2[locdf$tuberculosis > coords(roc1, "best")[["threshold"]]] <- 1

rocrpt0 <- reportROC(gold = as.factor(locdf$Y), predictor = locdf$tuberculosis, important = "se", plot = FALSE)

rocrpt1 <- reportROC(gold = as.factor(locdf$Y), predictor.binary = as.factor(locdf$tb1), important = "se", plot = FALSE)

rocrpt2 <- reportROC(gold = as.factor(locdf$Y), predictor.binary = as.factor(locdf$tb2), important = "se", plot = FALSE)

outdf <- data.frame(matrix(0, 2, 6))

outdf[[1]] <- c(0.35, round(coords(roc1, "best")[["threshold"]], 3))

outdf[1, 2] <- paste0(rocrpt0[["ACC"]], "\n(", rocrpt0[["ACC.low"]], " - ", rocrpt0[["ACC.up"]], ")")

outdf[1, 3] <- paste0(rocrpt1[["SEN"]], "\n(", rocrpt1[["SEN.low"]], " - ", rocrpt1[["SEN.up"]], ")")

outdf[1, 4] <- paste0(rocrpt1[["SPE"]], "\n(", rocrpt1[["SPE.low"]], " - ", rocrpt1[["SPE.up"]], ")")

outdf[1, 5] <- paste0(rocrpt0[["AUC"]], "\n(", rocrpt0[["AUC.low"]], " - ", rocrpt0[["AUC.up"]], ")")

outdf[1, 6] <- paste0(rocrpt0[["KAPPA"]], "\n(", rocrpt0[["KAPPA.low"]], " - ", rocrpt0[["KAPPA.up"]], ")")

outdf[2, 2] <- paste0(rocrpt0[["ACC"]], "\n(", rocrpt0[["ACC.low"]], " - ", rocrpt0[["ACC.up"]], ")")

outdf[2, 3] <- paste0(rocrpt2[["SEN"]], "\n(", rocrpt2[["SEN.low"]], " - ", rocrpt2[["SEN.up"]], ")")

outdf[2, 4] <- paste0(rocrpt2[["SPE"]], "\n(", rocrpt2[["SPE.low"]], " - ", rocrpt2[["SPE.up"]], ")")

outdf[2, 5] <- paste0(rocrpt0[["AUC"]], "\n(", rocrpt0[["AUC.low"]], " - ", rocrpt0[["AUC.up"]], ")")

outdf[2, 6] <- paste0(rocrpt0[["KAPPA"]], "\n(", rocrpt0[["KAPPA.low"]], " - ", rocrpt0[["KAPPA.up"]], ")")

colnames(outdf) <- c("阈值", "准确率", "灵敏度", "特异度", "AUC", "Kappa值")

regulartable(outdf) %>%

theme_vanilla() %>%

fontsize(size = 8, part = "header") %>%

fontsize(size = 7, part = "body") %>%

vline(i = NULL, j = c(1), border = fp_border(color="black"), part = "all") %>%

align(i = NULL, j = 1, align = "left", part = "all") %>%

align(i = NULL, j = 2:ncol(outdf), align = "center", part = "all") %>%

width(j = 1, width = 0.7) %>%

width(j = 2:ncol(outdf), width = 0.9) %>%

set_caption("AI评估结果的准确性指标")

```

## 对阳性病例的预测结果分析

&emsp;&emsp;本次研究主要关注活动性肺结核的预测准确率，但AI除了输出活动性肺结核的预测概率以外，还会输出陈旧性肺结核、肺炎、气胸、结节肿块、胸膜病变、心影增大等异常，我们将预测结果分为四类：活动性肺结核、陈旧性肺结核、其他异常、未见异常。针对不同的诊断结果，分别统计其预测比例，如表

\@ref(tab:disttbl3)所示。

```{r disttbl3, echo=FALSE, message=FALSE,results='asis',warning=FALSE}

outdf0 <- summarise(group_by(locdf[locdf$label %in% "tb", ], case, lab), num = length(patno), tb = sum(aires %in% "活动性肺结核"), tbrate = 0, oldtb = sum(aires %in% "陈旧性肺结核"), other = sum(aires %in% "其他异常"), norm = sum(aires %in% "未见异常"))

outdf <- rbind(as.data.frame(lapply(outdf0[outdf0$case %in% "确诊病例", ], FUN = function(X) ifelse(!inherits(X, "character"), sum(X), ifelse(grepl("病例", X[1]), X[1], "")))),

outdf0[outdf0$case %in% "确诊病例", ],

as.data.frame(lapply(outdf0[outdf0$case %in% "临床诊断病例", ], FUN = function(X) ifelse(!inherits(X, "character"), sum(X), ifelse(grepl("病例", X[1]), X[1], "")))),

outdf0[outdf0$case %in% "临床诊断病例", ],

as.data.frame(lapply(outdf0[outdf0$case %in% "疑似病例", ], FUN = function(X) ifelse(!inherits(X, "character"), sum(X), ifelse(grepl("病例", X[1]), X[1], "")))),

outdf0[outdf0$case %in% "疑似病例", ],

as.data.frame(lapply(outdf0, FUN = function(X) ifelse(!inherits(X, "character"), sum(X), ""))))

outdf$case[nzchar(outdf$lab)] <- ""

outdf$case[nrow(outdf)] <- "汇总"

outdf$tbrate <- paste0(format(outdf$tb / outdf$num * 100, digits = 1, nsmall = 1), "%")

outdf$abn <- paste0(format(100 - outdf$norm / outdf$num * 100, digits = 1, nsmall = 1), "%")

colnames(outdf) <- c("病例分类", "检测结果", "病例数", "活动性肺结核", "肺结核阳性率", "陈旧性肺结核", "其他异常", "未见异常", "异常率")

regulartable(outdf) %>%

theme_vanilla() %>%

fontsize(size = 8, part = "header") %>%

fontsize(size = 7, part = "body") %>%

hline(i = c(sum(outdf0$case %in% "确诊病例") + 1, sum(outdf0$case %in% c("确诊病例", "临床诊断病例")) + 2, nrow(outdf)-1), j = NULL, border = fp_border(width=1.5, color = gray(0.4)), part = "body") %>%

align(i = NULL, j = 1, align = "left", part = "all") %>%

align(i = NULL, j = 2:ncol(outdf), align = "center", part = "all") %>%

width(j = 1, width = 0.8) %>%

width(j = 2, width = 1.1) %>%

width(j = c(3:6, 9), width = 0.5) %>%

width(j = 7:8, width = 0.4) %>%

set_caption("各类别准确率")

```

## 和CT预测的对比

```{r sample4, include=FALSE}

ctlocdf1 <- cttb2df0[, c("patno", "tuberculosis")]

ctlocdf2 <- locdf[, c("patno", "tuberculosis")]

colnames(ctlocdf1) <- c("patno", "tuberculosis1")

colnames(ctlocdf2) <- c("patno", "tuberculosis2")

ctlocdf <- merge(ctlocdf1, ctlocdf2, all.x = TRUE)

ctlocdf <- ctlocdf[!is.na(ctlocdf$tuberculosis2), ]

cor1 <- cor.test(ctlocdf$tuberculosis1, ctlocdf$tuberculosis2, method = "spearman")

```

&emsp;&emsp;对于同时包含CT影像和CT定位片的病例，可计算各自预测肺结核的概率值，计算两种的相关系数，使用 Spearman 检验，得相关系数为

`r round(cor1$estimate[[1]], 3)`，P值为`r round(cor1$p.value[[1]], 3)`，可见存在显著相关，绘制散点图如图\@ref(fig:scatter1)所示，

```{r scatter1, fig.showtext = TRUE, fig.cap= 'CT影像和定位片的散点图', out.width='90%', fig.width=8, fig.asp=0.6, fig.align='center', echo=FALSE, message=FALSE}

plot(tuberculosis2~tuberculosis1, data = ctlocdf, xlab = "CT", ylab = "CT Loc")

```

&emsp;&emsp;由此可见，CT定位片在一定程度上可以描述CT中识别肺结核的影像特征。

## 和DR预测的对比

```{r sample5, include=FALSE}

drloc_dr <- drdf[drdf$patno %in% locdf$patno & drdf$label %in% "tb", ]

drloc_normal <- drdf[drdf$label %in% "normal", ]

drloc_loc <- locdf[locdf$patno %in% drdf$patno, ]

drloc_dr <- drloc_dr[order(drloc_dr$patno), ]

drloc_loc <- drloc_loc[order(drloc_loc$patno), ]

# identical(drloc_dr$patno, drloc_loc$patno)

drloc_all <- data.frame(patno = c(drloc_dr$patno, drloc_normal$patno), tbdr = c(drloc_dr$tuberculosis, drloc_normal$tuberculosis), tbloc = c(drloc_loc$tuberculosis, drloc_normal$tuberculosis), label = c(drloc_dr$label, drloc_normal$label), airesdr = c(drloc_dr$aires, drloc_normal$aires), airesloc = c(drloc_loc$aires, drloc_normal$aires), case = c(drloc_dr$case, drloc_normal$case), lab = c(drloc_dr$lab, drloc_normal$lab), stringsAsFactors = FALSE)

cor2 <- cor.test(drloc_dr$tuberculosis, drloc_loc$tuberculosis, method = "spearman")

drloc_all$Y <- 1

drloc_all$Y[drloc_all$label %in% "normal"] <- 0

roc1 <- roc(drloc_all$Y, drloc_all$tbdr)

roc2 <- roc(drloc_all$Y, drloc_all$tbloc)

```

&emsp;&emsp;由于CT定位片来自CT影像，在本研究中以肺部感染的CT影像为对照组，与对比健康人相比，性能可能被低估。我们从数据中提取既做了DR检查又做了CT检查且存在CT定位片的病例，共包含`r nrow(drloc_loc)`例，全为肺结核病例。计算两种检查的相关系数，使用

Spearman 检验，得相关系数为

`r round(cor2$estimate[[1]], 3)`，P值为`r round(cor2$p.value[[1]], 3)`，可见存在显著相关，绘制散点图如图\@ref(fig:scatter2)所示，

```{r scatter2, fig.showtext = TRUE, fig.cap= 'CT影像和定位片的散点图', out.width='90%', fig.width=8, fig.asp=0.6, fig.align='center', echo=FALSE, message=FALSE}

plot(x = drloc_dr$tuberculosis, y = drloc_loc$tuberculosis, xlab = "DR", ylab = "CT Loc")

```

&emsp;&emsp;基于DR和CT定位片分析的默认阈值，分别统计其对阳性病例的预测比例，如表 \@ref(tab:disttbl4)所示。

```{r disttbl4, echo=FALSE, message=FALSE,results='asis',warning=FALSE}

outdf0 <- summarise(group_by(drloc_all[drloc_all$label %in% "tb", ], case, lab), num = length(patno), tb1 = sum(airesdr %in% "活动性肺结核"), abnorm1 = sum(!airesdr %in% "未见异常"), tbrate1 = 0, abn1 = 0, tb2 = sum(airesloc %in% "活动性肺结核"), abnorm2 = sum(!airesloc %in% "未见异常"), tbrate2 = 0, abn2 = 0)

outdf <- rbind(as.data.frame(lapply(outdf0[outdf0$case %in% "确诊病例", ], FUN = function(X) ifelse(!inherits(X, "character"), sum(X), ifelse(grepl("病例", X[1]), X[1], "")))),

outdf0[outdf0$case %in% "确诊病例", ],

as.data.frame(lapply(outdf0[outdf0$case %in% "临床诊断病例", ], FUN = function(X) ifelse(!inherits(X, "character"), sum(X), ifelse(grepl("病例", X[1]), X[1], "")))),

outdf0[outdf0$case %in% "临床诊断病例", ],

as.data.frame(lapply(outdf0[outdf0$case %in% "疑似病例", ], FUN = function(X) ifelse(!inherits(X, "character"), sum(X), ifelse(grepl("病例", X[1]), X[1], "")))),

outdf0[outdf0$case %in% "疑似病例", ],

as.data.frame(lapply(outdf0, FUN = function(X) ifelse(!inherits(X, "character"), sum(X), ""))))

outdf$case[nzchar(outdf$lab)] <- ""

outdf$case[nrow(outdf)] <- "汇总"

outdf$tbrate1 <- paste0(format(outdf$tb1 / outdf$num * 100, digits = 1, nsmall = 1), "%")

outdf$abn1 <- paste0(format(outdf$abnorm1 / outdf$num * 100, digits = 1, nsmall = 1), "%")

outdf$tbrate2 <- paste0(format(outdf$tb2 / outdf$num * 100, digits = 1, nsmall = 1), "%")

outdf$abn2 <- paste0(format(outdf$abnorm2 / outdf$num * 100, digits = 1, nsmall = 1), "%")

colnames(outdf) <- c("病例分类", "检测结果", "病例数", "DR肺结核", "DR异常", "DR肺结核率", "DR异常率", "定位片肺结核", "定位片异常", "定位片肺结核率", "定位片异常率")

regulartable(outdf) %>%

theme_vanilla() %>%

fontsize(size = 8, part = "header") %>%

fontsize(size = 7, part = "body") %>%

hline(i = c(sum(outdf0$case %in% "确诊病例") + 1, sum(outdf0$case %in% c("确诊病例", "临床诊断病例")) + 2, nrow(outdf)-1), j = NULL, border = fp_border(width=1.5, color = gray(0.4)), part = "body") %>%

align(i = NULL, j = 1, align = "left", part = "all") %>%

align(i = NULL, j = 2:ncol(outdf), align = "center", part = "all") %>%

width(j = 1, width = 0.8) %>%

width(j = 2, width = 1.1) %>%

width(j = c(6:7, 10:11), width = 0.5) %>%

width(j = c(4:5, 8:9), width = 0.4) %>%

set_caption("各类别准确率")

```

&emsp;&emsp;当前DR和CT定位片有`r nrow(drloc_loc)`例共同的肺结核病例，DR分析时用来对照的健康人并无CT定位，所以无法估算这些人CT定位片的预测值，假设这些阴性样本存在CT定位片且其AI预测结果与DR预测结果完全一致，可以比较DR和CT定位片的ROC曲线，如图\@ref(fig:roc5)所示，

基于约登指计算DR的最佳阈值为 `r round(coords(roc1, "best")[["threshold"]], 3)`，计算得到的灵敏度为

`r round(coords(roc1, "best")[["sensitivity"]], 3)`，特异度为

`r round(coords(roc1, "best")[["specificity"]], 3)`。CT定位片的最佳阈值为

`r round(coords(roc2, "best")[["threshold"]][1], 3)`，计算得到的灵敏度为

`r round(coords(roc2, "best")[["sensitivity"]][1], 3)`，特异度为

`r round(coords(roc2, "best")[["specificity"]][1], 3)`。

```{r roc5, fig.showtext = TRUE, fig.cap= 'DR及CT定位片的ROC曲线', out.width='90%', fig.width=8, fig.asp=0.9, fig.align='center', echo=FALSE, message=FALSE}

plot(roc1, print.auc=TRUE, auc.polygon=TRUE,

grid.col=c("green", "red"), max.auc.polygon=TRUE,

auc.polygon.col="lightblue", print.thres=TRUE, xlim = c(1, 0))

plot(roc2, add = TRUE, col = "blue")

legend("bottomright", legend=c("CXR", "CT Localizer"),

col=c(par("fg"), "blue"), lwd=2)

```

&emsp;&emsp;基于不同的阈值，计算DR预测各准确性指标及其95\%置信区间，结果如表\@ref(tab:conftbl5)所示。

```{r conftbl5, echo=FALSE, message=FALSE,results='asis',warning=FALSE}

drloc_all$tb1 <- 0

drloc_all$tb1[drloc_all$tbdr > 0.35] <- 1

drloc_all$tb2 <- 0

drloc_all$tb2[drloc_all$tbdr > coords(roc1, "best")[["threshold"]]] <- 1

rocrpt0 <- reportROC(gold = as.factor(drloc_all$Y), predictor = drloc_all$tbdr, important = "se", plot = FALSE)

rocrpt1 <- reportROC(gold = as.factor(drloc_all$Y), predictor.binary = as.factor(drloc_all$tb1), important = "se", plot = FALSE)

rocrpt2 <- reportROC(gold = as.factor(drloc_all$Y), predictor.binary = as.factor(drloc_all$tb2), important = "se", plot = FALSE)

outdf <- data.frame(matrix(0, 2, 6))

outdf[[1]] <- c(0.35, round(coords(roc1, "best")[["threshold"]], 3))

outdf[1, 2] <- paste0(rocrpt0[["ACC"]], "\n(", rocrpt0[["ACC.low"]], " - ", rocrpt0[["ACC.up"]], ")")

outdf[1, 3] <- paste0(rocrpt1[["SEN"]], "\n(", rocrpt1[["SEN.low"]], " - ", rocrpt1[["SEN.up"]], ")")

outdf[1, 4] <- paste0(rocrpt1[["SPE"]], "\n(", rocrpt1[["SPE.low"]], " - ", rocrpt1[["SPE.up"]], ")")

outdf[1, 5] <- paste0(rocrpt0[["AUC"]], "\n(", rocrpt0[["AUC.low"]], " - ", rocrpt0[["AUC.up"]], ")")

outdf[1, 6] <- paste0(rocrpt0[["KAPPA"]], "\n(", rocrpt0[["KAPPA.low"]], " - ", rocrpt0[["KAPPA.up"]], ")")

outdf[2, 2] <- paste0(rocrpt0[["ACC"]], "\n(", rocrpt0[["ACC.low"]], " - ", rocrpt0[["ACC.up"]], ")")

outdf[2, 3] <- paste0(rocrpt2[["SEN"]], "\n(", rocrpt2[["SEN.low"]], " - ", rocrpt2[["SEN.up"]], ")")

outdf[2, 4] <- paste0(rocrpt2[["SPE"]], "\n(", rocrpt2[["SPE.low"]], " - ", rocrpt2[["SPE.up"]], ")")

outdf[2, 5] <- paste0(rocrpt0[["AUC"]], "\n(", rocrpt0[["AUC.low"]], " - ", rocrpt0[["AUC.up"]], ")")

outdf[2, 6] <- paste0(rocrpt0[["KAPPA"]], "\n(", rocrpt0[["KAPPA.low"]], " - ", rocrpt0[["KAPPA.up"]], ")")

colnames(outdf) <- c("阈值", "准确率", "灵敏度", "特异度", "AUC", "Kappa值")

regulartable(outdf) %>%

theme_vanilla() %>%

fontsize(size = 8, part = "header") %>%

fontsize(size = 7, part = "body") %>%

vline(i = NULL, j = c(1), border = fp_border(color="black"), part = "all") %>%

align(i = NULL, j = 1, align = "left", part = "all") %>%

align(i = NULL, j = 2:ncol(outdf), align = "center", part = "all") %>%

width(j = 1, width = 0.7) %>%

width(j = 2:ncol(outdf), width = 0.9) %>%

set_caption("DR评估结果的准确性指标")

```

&emsp;&emsp;基于不同的阈值，计算CT定位片预测各准确性指标及其95\%置信区间，结果如表\@ref(tab:conftbl6)所示。

```{r conftbl6, echo=FALSE, message=FALSE,results='asis',warning=FALSE}

drloc_all$tb1 <- 0

drloc_all$tb1[drloc_all$tbloc > 0.35] <- 1

drloc_all$tb2 <- 0

drloc_all$tb2[drloc_all$tbloc > round(coords(roc2, "best")[["threshold"]][1], 3)] <- 1

rocrpt0 <- reportROC(gold = as.factor(drloc_all$Y), predictor = drloc_all$tbloc, important = "se", plot = FALSE)

rocrpt1 <- reportROC(gold = as.factor(drloc_all$Y), predictor.binary = as.factor(drloc_all$tb1), important = "se", plot = FALSE)

rocrpt2 <- reportROC(gold = as.factor(drloc_all$Y), predictor.binary = as.factor(drloc_all$tb2), important = "se", plot = FALSE)

outdf <- data.frame(matrix(0, 2, 6))

outdf[[1]] <- c(0.35, round(coords(roc2, "best")[["threshold"]][1], 3))

outdf[1, 2] <- paste0(rocrpt0[["ACC"]], "\n(", rocrpt0[["ACC.low"]], " - ", rocrpt0[["ACC.up"]], ")")

outdf[1, 3] <- paste0(rocrpt1[["SEN"]], "\n(", rocrpt1[["SEN.low"]], " - ", rocrpt1[["SEN.up"]], ")")

outdf[1, 4] <- paste0(rocrpt1[["SPE"]], "\n(", rocrpt1[["SPE.low"]], " - ", rocrpt1[["SPE.up"]], ")")

outdf[1, 5] <- paste0(rocrpt0[["AUC"]], "\n(", rocrpt0[["AUC.low"]], " - ", rocrpt0[["AUC.up"]], ")")

outdf[1, 6] <- paste0(rocrpt0[["KAPPA"]], "\n(", rocrpt0[["KAPPA.low"]], " - ", rocrpt0[["KAPPA.up"]], ")")

outdf[2, 2] <- paste0(rocrpt0[["ACC"]], "\n(", rocrpt0[["ACC.low"]], " - ", rocrpt0[["ACC.up"]], ")")

outdf[2, 3] <- paste0(rocrpt2[["SEN"]], "\n(", rocrpt2[["SEN.low"]], " - ", rocrpt2[["SEN.up"]], ")")

outdf[2, 4] <- paste0(rocrpt2[["SPE"]], "\n(", rocrpt2[["SPE.low"]], " - ", rocrpt2[["SPE.up"]], ")")

outdf[2, 5] <- paste0(rocrpt0[["AUC"]], "\n(", rocrpt0[["AUC.low"]], " - ", rocrpt0[["AUC.up"]], ")")

outdf[2, 6] <- paste0(rocrpt0[["KAPPA"]], "\n(", rocrpt0[["KAPPA.low"]], " - ", rocrpt0[["KAPPA.up"]], ")")

colnames(outdf) <- c("阈值", "准确率", "灵敏度", "特异度", "AUC", "Kappa值")

regulartable(outdf) %>%

theme_vanilla() %>%

fontsize(size = 8, part = "header") %>%

fontsize(size = 7, part = "body") %>%

vline(i = NULL, j = c(1), border = fp_border(color="black"), part = "all") %>%

align(i = NULL, j = 1, align = "left", part = "all") %>%

align(i = NULL, j = 2:ncol(outdf), align = "center", part = "all") %>%

width(j = 1, width = 0.7) %>%

width(j = 2:ncol(outdf), width = 0.9) %>%

set_caption("CT定位片评估结果的准确性指标")

```

&emsp;&emsp;我们使用 delong 检验来检验 DR 模型和 CT 定位片模型的 ROC 差异，如表\@ref(tab:delong1)所示。

```{r delong1, echo=FALSE, message=FALSE, results='asis',warning=FALSE}

test1 <- roc.test(roc1, roc2, method = "delong", alternative = "two.sided", paired = FALSE)

test2 <- roc.test(roc1, roc2, method = "delong", alternative = "less", paired = FALSE)

outdf <- data.frame(matrix(0, 2, 4))

outdf[[1]] <- c("two.sided", "less")

outdf[1, 2] <- round(test1$statistic[["D"]], 3)

outdf[1, 3] <- round(test1$parameter[["df"]], 2)

outdf[1, 4] <- round(test1$p.value, 4)

outdf[2, 2] <- round(test2$statistic[["D"]], 3)

outdf[2, 3] <- round(test2$parameter[["df"]], 2)

outdf[2, 4] <- round(test2$p.value, 4)

colnames(outdf) <- c("h1", "D", "df", "P Value")

regulartable(outdf) %>%

theme_vanilla() %>%

fontsize(size = 8, part = "header") %>%

fontsize(size = 7, part = "body") %>%

align(i = NULL, j = 1, align = "left", part = "all") %>%

align(i = NULL, j = 2:ncol(outdf), align = "center", part = "all") %>%

width(j = 1, width = 0.55) %>%

width(j = 2:ncol(outdf), width = 0.63) %>%

set_caption(" delong 检验")

```

&emsp;&emsp;由此可见， DR

模型的AUC

低于

CT

定位片模型，但差异并不显示。需要注意的是，当前CT定位片的AUC值可能被高估，因为其中阴性样本的预测值来自DR数据的预测，实际上阴性样本并不存在CT定位片。不过基于经验，有理由相信AI对阴性样本的预测能力较强，所以基于CT定位片的预测具有潜力，值得深入研究。
